# The well-being of Jewish college students in the United States

**DOI:** 10.1371/journal.pone.0357478

**Published:** 2026-09-11

**Authors:** Simone Ispa-Landa, Avital Strauss, Eleanor Ostroff, Harrison Garcia, Yonatan Lean, Jonathan Kfir, Gregory E. Miller

**Affiliations:** 1 Institute for Policy Research, School of Education and Social Policy, and Department of Sociology, Northwestern University, Evanston Illinois, United States of America; 2 Institute for Policy Research, Northwestern University, Evanston IL and Brown University, Providence Rhode Island, United States of America; 3 Northwestern University Feinberg School of Medicine, Chicago Illinois, United States of America; 4 University of California, Berkeley, California, United States of America; 5 Institute for Policy Research and Department of Psychology, Northwestern University, Evanston Illinois, United States of America; University of Durham: Durham University, UNITED KINGDOM OF GREAT BRITAIN AND NORTHERN IRELAND

## Abstract

Since October 7, 2023, protests related to the Israel-Hamas conflict have occurred at colleges and universities across the United States. At some institutions these demonstrations have prompted civil rights investigations, interruptions to research funding, and/or revisions to campus speech policies. Government and university leaders have often cited concerns about Jewish students’ well-being as the impetus for these interventions. However, it is unclear to what extent these events, and the broader campus atmosphere around Judaism and Israel, have affected the well-being of Jewish students. Thus, we surveyed 3418 US college students from January-June 2025 (mean age = 20; 57% women; 68% White; 17% Asian; 12% Hispanic; 3% Black). Students completed questionnaires regarding demographics, anxiety, depression, loneliness, flourishing, sense of belonging, and campus atmosphere. The preregistered goals were to (1) evaluate Jewish students’ well-being relative to peers with other religious identities and (2) examine how strongly Jewish students’ well-being was related to sense of belonging on campus and perceptions of institutional atmosphere toward Israel and Judaism. With regard to the first goal, 47% of Jewish students reported being somewhat or very concerned about campus antisemitism**.** Nonetheless**,** on measures of depression, anxiety, loneliness, and flourishing, they reported similar well-being compared with students of other religions, and better well-being compared with students who did not endorse a religious identity. Similar patterns were observed in a large survey of US college students performed from 2016–2022. With regard to the second goal, Jewish students’ well-being in 2025 was positively associated with both perceiving a less hostile campus atmosphere and experiencing a greater sense of campus belonging. However, sense of belonging explained 2–4 times more variability in outcomes. These findings indicate that despite prevalent concerns about anti-semitism, Jewish students had similar or better well-being than peers 15–20 months after the hostilities of October 7, 2023.

## Introduction

The well-being of US college students has declined markedly over the past decade. Nationwide survey data indicate that in 2024, nearly 40% of US college students reported moderate-to-severe symptoms of depression, and over 50% reported high levels of loneliness [1]. At this life stage, lower well-being, whether reflected in mental health problems or social disconnection, is prospectively associated with higher suicide risk, worse cardiometabolic health, difficulties in interpersonal relationships, and struggles in the labor market [2–4].

In response to these trends, government officials and university administrators have become increasingly focused on student well-being and how it is influenced by features of the campus atmosphere [5,6]. Concerns about the well-being of Jewish students became especially prominent in the aftermath of October 7, 2023 [7–9], when Hamas led an attack on Israel that killed ~1,200 Jews and took another ~200 people as hostages. Within several weeks of the attacks, demonstrations and encampments began on multiple campuses across the US, protesting the actions of the Israeli military and in some cases questioning Israel’s legitimacy as a Jewish state. During this period, some college students reported experiencing a broader anti-Jewish sentiment on campus: in one survey, 33% of students said that faculty on their campus had “*promoted antisemitism or fueled a learning environment that is hostile to Jews*” [10]. These events coincided with a marked increase in the rate of antisemitic incidents, both in the US and globally [11]. More concretely, Jews comprised 2% of the US population in 2024, but were the targets of 18% of all hate crimes, a per-capita victimization rate more than 2.5-fold higher than any other group [12].

When students returned to campus in fall 2024, many colleges and universities announced significant changes in policy [9,13]. These changes included new restrictions on protests, expanded systems for reporting antisemitic harassment and discrimination, and mandatory trainings about antisemitism. In parallel, federal agencies were launching civil rights investigations and freezing research funding at multiple universities [14]. By early 2025, the freezes had disrupted more than 1800 studies funded by the National Institutes of Health [15]. Many of the affected institutions responded to the resulting budget shortfalls by laying off staff, freezing new hiring, and trimming fringe benefits.

At times, both university leaders and government officials pointed to concerns about the well-being of Jewish college students as the impetus for these interventions [7,8,16,17]. For instance, concluding that “*the bias experienced by Jews and Israelis led to an increase in stress of survey respondents and resulted in negative impacts on mental health and overall well-being*,” a UCLA Task Force on Antisemitism recommended changing multiple policies around campus speech. These recommendations included new time, place, and manner restrictions on protests, and a prohibition on departmental statements about “*public matters that do not affect the university’s core mission*” [16].

Although it is reasonable to hypothesize that exposure to campus antisemitism might undermine Jewish students’ well-being, the extent to which it does so remains an open question. Research with other populations who have been persistently mistreated and/or stigmatized, e.g., Black Americans and sexual/gender minorities, has identified a host of protective factors which can buffer against the adverse consequences of identity-based stressors [18–21]. These protective factors include positive ingroup identity, meaningful social connections, and particular coping styles, and they could plausibly serve as buffers for Jewish students as well.

With that said, there is a dearth of empirical research in this domain. Two studies considered how experiences of antisemitism related to the well-being of Jewish college students after October 7, 2023 [22,23]. However, neither sought to quantify the well-being of Jewish students relative to other students or relative to their own well-being before the attacks. In a survey conducted in Germany in early 2024, Jewish university students reported higher levels of anxiety, depression, and stress compared with non-Jewish peers [24]. In the US context, the most germane results are from a working paper by Hersh and Lyss [25], which describes a panel study of more than 500 college students. They report that in the months immediately following October 7, 2023, 25% of Jewish students rated their mental health as “*poor*” vs. 13% of non-Jewish students. When students were re-interviewed approximately 6 months later, these figures declined to 15% and 9%, leading the authors to conclude that “*Jewish students at the start of the war had a temporary decline in self-reported mental health, but then returned to more typical responses*” [25].

Our goal in this study was to gather further evidence in this domain. We conducted a nationwide survey of US college students, using standardized instruments to quantify well-being along multiple dimensions, including depression, anxiety, loneliness, and flourishing. The survey began in January 2025, approximately 8 months after the peak of campus protests, and ended in June 2025. To contextualize the results of the survey, we also present secondary analyses of a nationwide study of US college students [26]. From 2016–2022, this survey collected data about religious identity and administered the same measures of well-being as our study, providing historical context for our 2025 results.

Drawing on research with other student populations who have been stigmatized and/or mistreated [5,6], we also considered whether well-being would covary with campus belonging (feeling valued, connected, and accepted) and perceived campus atmosphere (whether it is seen as hostile toward one’s group). Both of these traits are key predictors of minority students’ well-being [27,28]. This question is a timely one, as a number of campus initiatives aim to improve students’ well-being by either fostering a greater sense of belonging (e.g., through affinity groups) or by changing the campus atmosphere (e.g., through anti-discrimination policies or policies aimed at speech and/or protest) [29].

## Methods & materials

The study design, hypotheses, and analysis plan were pre-registered on January 29, 2025 at https://osf.io/82ube/overview. At the time pre-registration was filed, we had begun surveying participants but had not reviewed or analyzed any responses. All data presented here are included as Online Supporting Information (except for information regarding each student’s college or university, which was withheld to ensure privacy.) HMS data are public and can be acquired from the study investigators at https://healthymindsnetwork.org.

### Participants

To be eligible, students had to be ≥ 18 years and enrolled full-time at a college or university in the US. We used social media, student organizations, and word-of-mouth to publicize the study. Interested parties were directed to a study website, where they answered questions about study eligibility criteria and provided an e-mail address with an.edu suffix. To verify their affiliation with a college or university, we sent a follow-up message to that account, containing a hyperlink to the consent form and survey materials. Students received $15 for participating. For each person they referred to the study, students received an additional $10, up to a maximum of four referrals. All students gave consent before participating, and Northwestern University’s Institutional Review Board approved the protocol (STU00222672).

### Protocol and measures

The study was administered by a survey research firm (Forthright) using an online platform. After providing consent, students answered questions about demographics, well-being, and campus experiences. The full text of the survey can be accessed here: https://osf.io/qh69b. Unless otherwise specified, each instrument was administered to all participants. Two students did not complete the full survey; their responses were excluded from statistical analysis.

To characterize religious identity, we asked, “*Which of the following religions, if any, do you identify with (Select all that apply)?*” The options were Protestantism, Catholicism, Other Christian, Judaism, Latter Day Saints, Islam, Buddhism, Hinduism, Other Religion Not Specified, and None. To simplify hypothesis testing, we assigned each student to one of four groups. The Jewish group contained anyone who selected Judaism (n = 2006), even if they also endorsed another religion. We refer to these respondents as “Jewish students” hereafter. However, we acknowledge the question’s framing treats Judaism as a religious identity, and therefore may have led to the mis-classification of individuals who identify as ethnically but not religiously Jewish. The Christian group contained those who selected Protestantism (n = 89), Catholicism (n = 424), or Other Christian (n = 242). The Other Religion group contained those who selected LDS (n = 6), Islam (n = 61) Buddhism (n = 62), Hinduism (n = 99), or Other religion not specified (n = 44). Students who chose “*None*” were placed in a group labelled No Religious Identity (n = 475).

We characterized well-being along four dimensions, using validated instruments to assess depression (Patient Health Questionnaire-8 or PHQ-8 [30]), anxiety (Generalized Anxiety Disorder-7 Scale or GAD-7 [31]), and loneliness (Brief Loneliness Scale [32]), along with flourishing, a concept that encompasses meaning in life, success in relationships, and optimism about the future (Flourishing Scale [33]). We omitted the PHQ’s question about suicidality because we lacked resources to follow-up with participants who endorsed it. Other surveys have omitted this item for the same reason and found the scale remains valid [30]. All four instruments displayed strong internal consistency, with Cronbach’s α from.77 (loneliness) to.89 (anxiety).

To understand what contributes to variation in Jewish students’ well-being, we measured their sense of belonging on campus and perceptions of the atmosphere towards Judaism and Israel. On the Sense of Belonging Scale (Cronbach’s α = .85), students indicated how much they agreed with eight statements, e.g., “*I feel connected with other students at my school*” [34]. Campus atmosphere was assessed with four questions drawn from the panel study mentioned in the Introduction, focusing on Jewish students’ experiences following October 7, 2023 [25]. First, on a four-point scale from “*not at all*” to “*very much*,” students indicated how concerned they were about antisemitism on their campus. Second, on five-point scales ranging from “*strongly agree*” to “*strongly disagree*,” students indicated how much they agreed with these statements: “*In order to fit in on my campus, I feel the need to hide that I am Jewish,*” “*People will judge me negatively if I participate in Jewish activities on campus*,” and “*On my campus, Jewish students pay a social penalty for supporting the existence of Israel as a Jewish state*.” (Only students who identified as Jewish were administered the latter 3 items.) After converting responses into z-scores, we summed them to form an atmosphere composite (Cronbach’s α = .85).

### Healthy Minds Study

To historically contextualize the results of the 2025 survey, we conducted parallel analyses of publicly available data from the Healthy Minds Study (HMS). Over the past 15 years, HMS has surveyed nearly a million college and university students across the US [1,26], administering the same measures of well-being as our 2025 survey. (Except for the loneliness items, which were not given until the 2020/21 academic year.) Each year, participating institutions offer a random subset of students the chance to participate in HMS. Through the 2021/22 academic year the survey asked students, “*What is your religious affiliation*?” but discontinued this item in subsequent years. We used students’ responses to assign them to one of four religion groups in a manner parallel to our 2025 survey. Though HMS items about student demographics varied somewhat over time, we used the available data to develop covariates that closely mirrored the 2025 survey. We analyzed HMS data spanning the 2016/17–2021/22 academic years, excluding respondents with incomplete data on religion, covariates, or well-being indicators.

### Statistical analyses

The first pre-registered aim was to characterize the well-being of Jewish students relative to peers. To accomplish this, we estimated separate analyses of covariance for each dimension of well-being. The predictors were the four-group religion variable and a set of covariates, described below. In models where the omnibus test of religious group was significant, we followed-up with planned contrasts comparing Jewish students to other religion categories. The second pre-registered aim was to understand how campus atmosphere and belonging related to Jewish students’ well-being. To accomplish this, we estimated linear regression models for each well-being dimension, with the covariates entered as predictors on step 1 and either atmosphere or belonging entered on step 2.

Primary models included a set of covariates specified *a priori* in the pre-registration. They were age (in years), gender identity (to harmonize across studies, this construct was simplified into a binary variable reflecting whether the participant identified as a woman), racial/ethnic identity (modeled using a series of dummy variables, such that White was the reference category), and field of study (modeled using a series of dummy variables, such that humanities was the reference category). In robustness analyses we further adjusted models for neuroticism, a personality trait that predisposes individuals to experience persistent distress. Neuroticism was assessed with a two-item scale widely used in large surveys [35]. In additional robustness checks we adjusted for the academic selectivity of the institution each student attended, using the school’s mean score on the Scholastic Aptitude Test (SAT), drawn from data gathered by Opportunity Insights [36]. To determine whether the sample’s nested structure (students within institutions) biased parameter estimates, we estimated the intraclass correlation for each well-being measure. The coefficients were quite small in magnitude (all < .05), indicating minimal within-institution clustering of well-being. Nevertheless, as a further check on biases related to clustering, we re-modeled the associations using Generalized Estimating Equations, specifying institution as a nesting factor and an exchangeable covariance matrix.

### Departures from pre-registered plans

We departed from the study’s pre-registered design in three respects. First, we expanded the sample and changed eligibility criteria partway through data collection. Specifically, the pre-registration proposed a sample of 2,500 based on power analyses and available funding. Shortly after reaching that target, we received additional funding to identify sources of well-being variation among Jewish students. To facilitate that goal, we expanded the sample to 3,500 and required that all further participants identify as Jewish. Second, the pre-registered analysis plan entailed comparing Jewish students in our survey with Jewish students in HMS from 2016–2020. We later realized this strategy made sub-optimal use of HMS. To best contextualize the results of our survey, we opted to conduct parallel analyses in HMS. Third, we proposed analyses of whether a strong Jewish identity enhances well-being. However, there was too little variability in students’ identity to permit this analysis.

## Results

A total of 3418 students completed the survey between January 25, 2025 and June 13, 2025. They attended 331 different colleges and universities in 39 different states. The students were concentrated in regions of the country with larger Jewish populations, including the Northeast, Florida, and California (S1a and S1b in S1 Fig).

Table 1 presents the sample characteristics. More than 99% of students attended 4-year institutions, the majority of which (59.2%) were publicly funded. Based on an established five-level classification system [36], 25.2% of students attended institutions in the highest tier of selectivity (“elite” schools). An additional 43.1% attended institutions in the next tier (“highly selective” schools). Jewish students differed from those endorsing other religious identities on all characteristics (p’s < .001). On demographic indicators, they were older and more likely to identify as women and as White. On educational characteristics, they were more likely to be majoring in social science or humanities disciplines and to attend private universities. The mean SAT scores of students’ institutions were fairly similar across groups. The exception was schools that Christian students attended, which had mean scores 25–30 points lower (p’s < .001). As a group, the Jewish students in the sample identified strongly with Judaism and Israel: 71.6% considered themselves as “*Zionist*” versus 15.2% as “*Non-Zionist*” and 13.2% as “*Unsure*”. Moreover, 82.2% strongly agreed with the statement that “*being Jewish is very important to me*.”

**Table 1:** Characteristics of main sample, surveyed in 2025.

	Full Sample (n = 3418)	Jewish Students (n = 2006)	Christian Students (n = 697)	Other Religion (n = 240)	No Religion (n = 475)
	Mean (SD) or N (%)	Mean (SD) or N (%)	Mean (SD) or N (%)	Mean (SD) or N (%)	Mean (SD) or N (%)
Age, years	20.3 (2.1)	20.4 (2.4)	20.0 (1.5)	20.2 (1.6)	20.1 (1.5)
Identifies as man	1389 (40.6%)	761 (37.9%)	307 (44.0%)	127 (52.9%)	194 (40.8%)
Identifies as woman	1944 (56.9%)	1186 (59.1%)	384 (55.1%)	110 (45.8%)	264 (55.6%)
Identifies as White	2334 (68.3%)	1741 (86.8%)	348 (49.9%)	25 (10.4%)	220 (46.3%)
Identifies as Asian	577 (16.9%)	46 (2.3%)	138 (19.8%)	179 (74.6%)	214 (45.1%)
Identifies as Hispanic	411 (12.0%)	153 (7.6%)	195 (28.0%)	10 (4.2%)	53 (11.2%)
Identifies as Middle Eastern	264 (7.7%)	218 (10.9%)	16 (2.3%)	16 (6.7%)	14 (2.9%)
Identifies as Black	121 (3.5%)	25 (1.2%)	57 (8.2%)	16 (6.7%)	23 (4.8%)
Major – STEM field	1297 (37.5%)	650 (32.4%)	297 (42.6%)	137 (57.1%)	213 (44.8%)
Major – Social sciences	816 (23.9%)	558 (27.8%)	122 (17.5%)	27 (11.3%)	109 (22.9%)
Major – Humanities	428 (12.5%)	308 (15.4%)	58 (8.3%)	15 (6.3%)	47 (9.9%)
Public college/university *	2023 (59.2%)	1109 (55.5%)	424 (62.1%)	173 (72.7%)	317 (67.3%)
Mean SAT college/university *	1270.4 (125.1)	1275.3 (118.6)	1248.9 (133.9)	1283.0 (137.8)	1272.3 (130.3)

***Note***. Values for racial/ethnic identity exceed 100%, because students can endorse multiple identities. On all characteristics, Jewish students differ from at least one other group at *p* < .001. * For public college/university, n = 3391 for mean SAT, n = 3083.

### Well-being differences by religion

**Fig 1:**
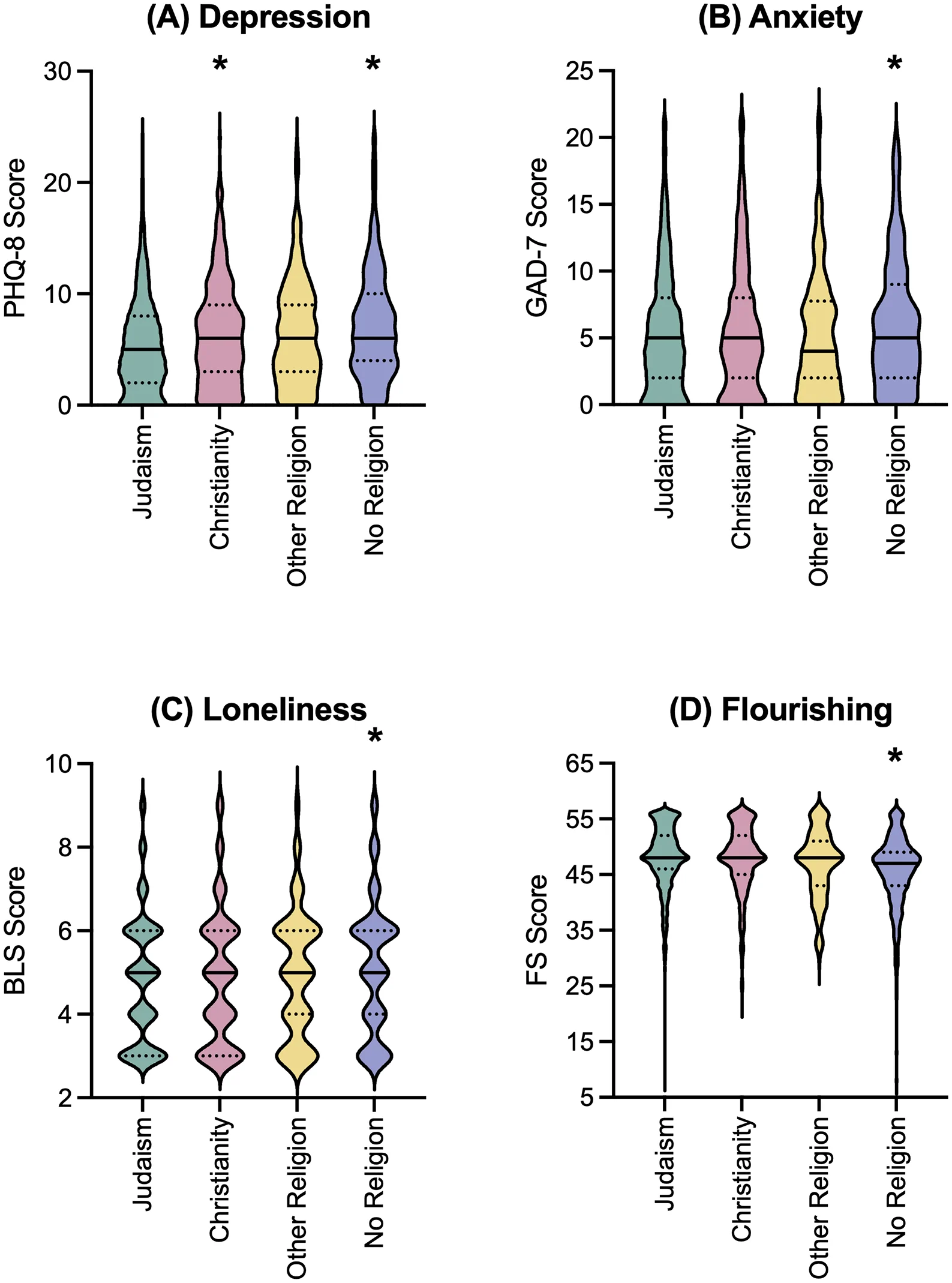
Distribution of well-being indicators in 2025 survey, stratified by religious identity. The plots depict the distribution of observed values for each dimension of well-being. The solid horizontal bars are the group’s median value, and the dashed horizontal bars are the 25^th^ and 75^th^ percentile of the group’s distribution. Asterisk indicates group significantly differs from Jewish students at *p* < .005.

In primary models, Jewish students reported similar well-being compared to their peers (Fig 1). There were several exceptions to this general pattern: Jewish students reported better well-being on all four dimensions compared to students who endorsed no religious identity, and lower depressed mood compared to Christian students (p’s < .001). Although statistically significant, these differences were small in magnitude (Table 2), e.g., < 1.5 points on the PHQ-8, which ranges from 0–24, and < 2.0 points on the Flourishing Scale, which ranges from 8–64.

**Table 2:** Results of pre-registered comparisons between Jewish students and those of other religions.

	Christian – Jewish *Contrast Estimate (95% CI)*	Other Religion – Jewish *Contrast Estimate (95% CI)*	No Religion – Jewish *Contrast Estimate (95% CI)*
PHQ-8 Depression (possible range 0–24)	0.79 (0.37, 1.21) *	0.54 (−0.17, 1.25)	1.46 (0.96, 1.97) *
GAD-7 Anxiety (possible range 0–21)	0.36 (−0.07, 0.80)	−0.07 (−0.80, 0.66)	0.77 (0.25, 1.28) *
Loneliness (possible range 3–9)	0.05 (−0.09, 0.20)	−0.01 (−0.26, 0.23)	0.29 (0.12, 0.47) *
Flourishing (possible range 8–64)	0.19 (−0.37, 0.74)	0.27 (−0.66, 1.20)	−1.70 (−2.35, −1.04) *
Moderate/Severe Depression (PHQ-8 score ≥ 10)	6.2% (2.5%, 9.9%) *	7.2% (1.0%, 13.3%) *	10.0% (5.6%, 14.4%) *
Moderate/Severe Anxiety (GAD-7 score ≥ 10)	2.7% (−0.9%, 6.4%)	−1.2% (−7.3%, 5.0%)	5.5% (1.1%, 9.9%) *

***Notes***. Contrast estimates and confidence intervals are derived from analyses of covariance, and are adjusted for age, gender, racial/ethnic identity, and field of study. Positive estimates indicate the comparison group has higher values on the dimension relative to Jewish students; negative estimates indicate the opposite. * denotes 95% confidence interval that excludes the null value of 0.

**Fig 2:**
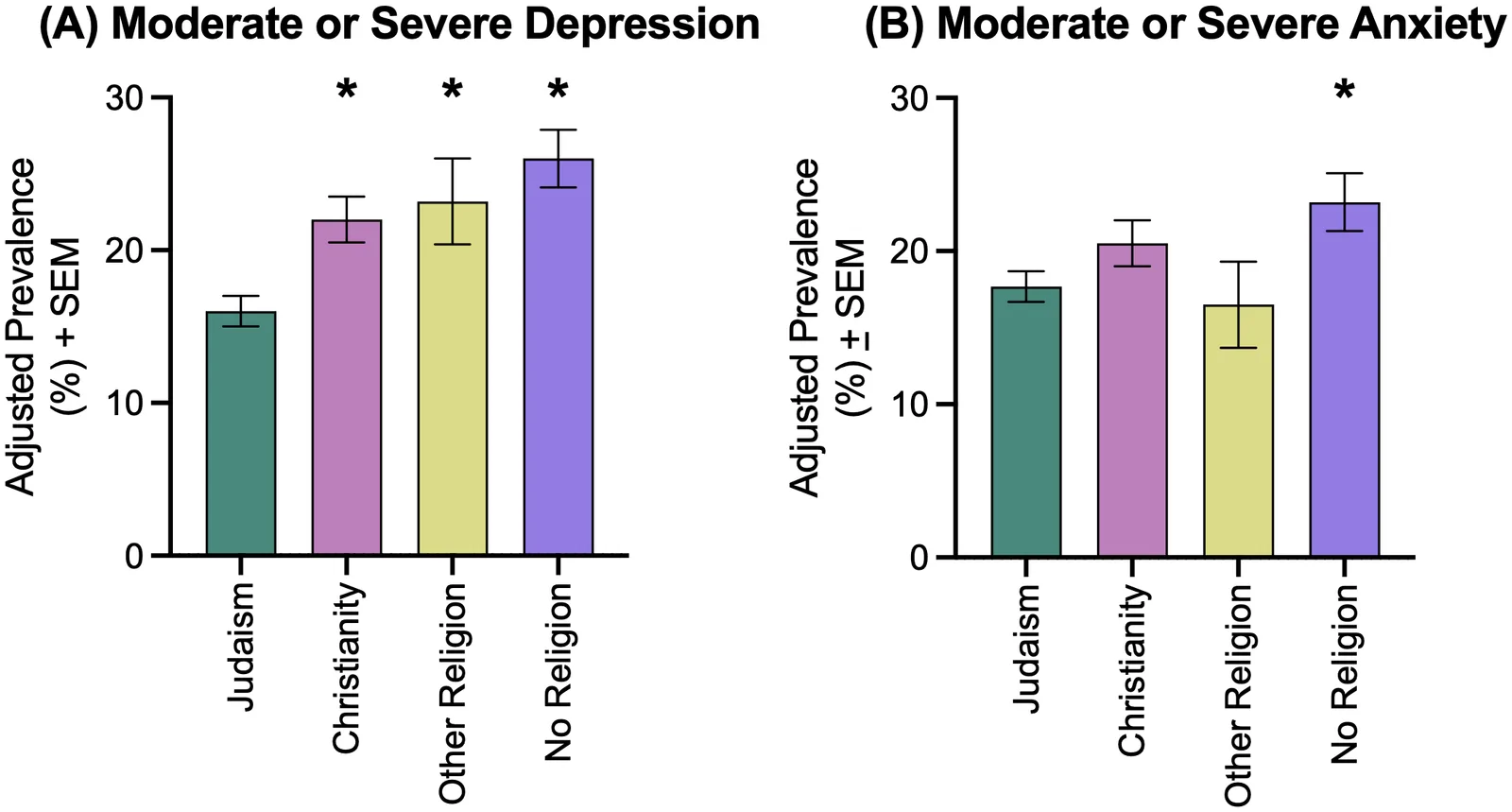
Clinically meaningful distress in 2025 survey, stratified by religious identity. Each figure depicts the estimated prevalence of moderate or severe distress and the standard error around that mean. Values are adjusted for age, gender, racial/ethnic identity, and field of study. Asterisk indicates group significantly differs from Jewish students at *p* < .02 or lower.

To further clarify the meaning of these patterns, we classified the severity of students’ PHQ-8 and GAD-7 scores, using established thresholds of 10 to define cases with moderate or severe distress. (These analyses were not pre-registered and should be viewed as exploratory.) In PHQ-8 models, Jewish students reported significantly lower rates of moderate/severe depression compared with students in all three other religious categories (p’s < .001; Fig 2a). The adjusted differences in prevalence were −6.2% (vs. Christian students), −7.2% (vs. students of other religions), and −10.0% (vs. students who did not endorse a religious identity). In GAD-7 models, Jewish students reported significantly lower rates of moderate/severe anxiety than students who did not endorse a religious identity (adjusted difference = −5.5%; p < .001) but did not differ from students in the Christian or Other Religion groups (Fig 2b).

In sensitivity analyses where the models were further adjusted for the personality trait of neuroticism and selectivity of the institution each student attended, the findings were highly similar (S1 and S2 Tables). Also, when we remodeled associations using Generalized Estimating Equations to account for the sample’s nested structure, the same patterns were evident (S3 Table).

To provide context for interpreting these results, we conducted parallel analyses of 507,010 HMS participants surveyed between 2016 and 2022, using the same well-being dimensions and covariates (S4 Table). Similar to the patterns in our 2025 survey, Jewish students reported significantly better well-being compared with students No Religion group (Table 3). However, unlike in the 2025 survey, Jewish students reported lower well-being than Christian students and better well-being than students in the Other Religion group. Comparing values from our survey with those from HMS, there was no indication that the

**Table 3:** Results of pre-registered comparisons between Jewish students and those of other religions in Healthy Minds Study, surveyed between 2016 and 2022.

	Christian – Jewish *Contrast Estimate (95% CI)*	Other Religion – Jewish *Contrast Estimate (95% CI)*	No Religion – Jewish *Contrast Estimate (95% CI)*
PHQ-8 Depression (possible range 0–24)	−0.43 (−0.56, −0.31) *	1.16 (1.02, 1.29) *	1.32 (1.20, 1.45) *
GAD-7 Anxiety (possible range 0–21)	−0.47 (−0.58, −0.35) *	0.69 (0.56, 0.81) *	0.51 (0.40, 0.62) *
Loneliness (possible range 3–9)	−0.25 (−0.32, −0.17) *	0.15 (0.07, 0.23) *	0.02 (−0.05, 0.10)
Flourishing (possible range 8–64)	0.87 (0.63, 1.10) *	−0.65 (−0.91, −0.39) *	−2.79 (−3.03, −2.56) *
Moderate/Severe Depression (PHQ-8 score ≥ 10)	−2.8% (−3.8%, −1.8%) *	8.2% (7.1%, 9.3%) *	8.8% (7.8%, 9.8%) *
Moderate/Severe Anxiety (GAD-7 score ≥ 10)	−2.6% (−3.5%, −1.7%) *	5.6% (4.6%, 6.5%) *	4.2% (3.3%, 5.1%) *

***Notes***. Contrast estimates and confidence intervals are derived from analyses of covariance, and are adjusted for age, gender, racial/ethnic identity, and field of study. Positive estimates indicate the comparison group has higher values on the dimension relative to Jewish students; negative estimates indicate the opposite. * denotes 95% confidence interval that excludes the null value of 0.

well-being of Jewish students was worse in 2025 compared with the period from 2016–2022. In fact, like students of other religious identities, those who identified as Jewish reported markedly better well-being across dimensions in 2025, relative to the pandemic years of 2020–2022. The same pattern was evident for moderate/severe depression and anxiety (S2 and S3 Figs).

### Sources of variation in Jewish student well-being

S4 Fig shows Jewish students’ perceptions of campus atmosphere. When asked how concerned they were about campus antisemitism, 23% replied “*very much*,” 24% “*somewhat*,” 30% “*a little*,” and 23% “*not at all*.” When asked to say how much they agreed with the statement that “*Jewish students pay a penalty for supporting the existence of Israel as a Jewish state,*” 18% replied “*strongly agree*” and 28% replied “*agree*.” A small proportion replied “*strongly agree*” to items regarding the need to hide their Jewish identity (6%) and being judged negatively for participating in Jewish activities (8%).

In adjusted regression models with Jewish students, perception of campus atmosphere was associated with all dimensions of well-being (S5 Table). For each 1 SD increase in perceived hostility toward Judaism/Israel, students reported 0.56 higher depression scores, 0.81 higher anxiety scores, 0.26 higher loneliness scores, and 0.66 lower flourishing scores. Net of covariates, campus atmosphere explained 4%−8% of the variation in well-being dimensions among Jewish students.

**Fig 3:**
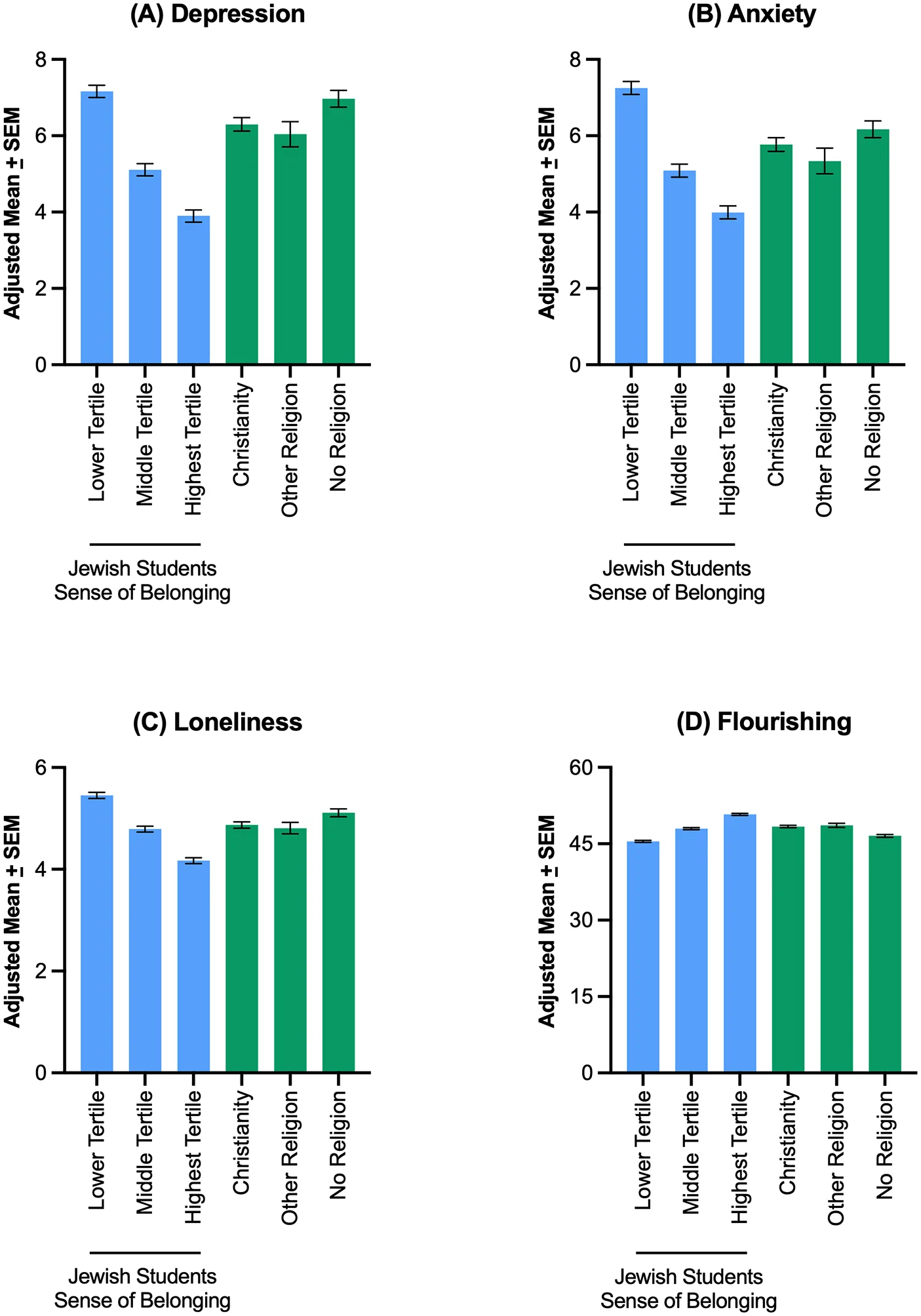
Jewish students’ well-being in 2025 survey stratified by their sense of belonging. Values for other religious groups are shown for comparison, without stratification. All values are adjusted for age, gender, racial/ethnic identity, and field of study.

In adjusted regression models, sense of belonging was associated with all four dimensions of well-being (S6 Table). For each 1 SD higher belonging score, students reported 1.50 lower depression scores, 1.51 lower anxiety scores, 0.63 lower loneliness scores, and 2.38 higher flourishing scores. Net of covariates, belonging explained 10%−16% of the variation in well-being among Jewish students (Fig 3). To understand the impact of October 7, 2023 on belonging, we asked Jewish students how much their sense of belonging on campus had changed since the hostilities began. 11% reported that it had “*declined a lot*” and 20% said that it had “*declined a little*.”

When perceptions of campus atmosphere and sense of belonging were simultaneously entered into the regression models, only the latter remained consistently associated with well-being (S7 Table). In these models, campus atmosphere explained <1% of variation in the well-being of Jewish students, whereas sense of belonging explained 8%−16%. The results were unchanged when these models were further adjusted for institutional selectivity.

## Discussion

In a 2025 survey of 3,418 college students from across the United States, Jewish respondents reported similar levels of well-being compared to peers who identified with other religions, and better well-being than students who endorsed no religious identity. These patterns were consistent across multiple dimensions of well-being, including anxiety, depression, loneliness, and flourishing. Interestingly, Jewish students’ comparatively similar levels of well-being coexisted with highly prevalent concerns about campus atmosphere: 77% of Jewish students reported at least some concerns about antisemitism on their campus, and 47% anticipated social penalties for supporting Israel as a Jewish state.

These findings largely mirror patterns evident in national data collected by HMS from 2016–2022. During those years Jewish students also reported consistently better well-being than peers with no religious identity. In both surveys Jewish students also reported slightly better well-being than students who identified as Buddhist, Hindu, Muslim, and LDS. (These differences were more often statistically significant in HMS because of its massive sample, but from a clinical perspective the size of the contrast estimates was similar across studies.) However, the studies yielded divergent results when comparing Jewish and Christian students. In the 2025 survey, Jewish students had lower depression, but were similar to Christian students on other dimensions. In HMS surveys from 2016–2022, Christian students had higher well-being across dimensions, although from a clinical perspective the differences were modest in size. We do not have a ready explanation for this discrepancy.

In interpreting these results, it is important to note that our survey entered the field in January 2025, approximately 8 months after the peak of campus protests during the 2023/24 academic year. As such, it did not capture Jewish students’ well-being as the demonstrations and encampments were occurring or in their immediate aftermath. This timeline may explain why our results diverge from those of the survey in Germany [24] and the panel study [25] described in the Introduction. The latter observed that in the months immediately following the October 7, 2023 attacks, American Jewish students were more likely to rate their mental health as “*poor*” compared with peers (25% vs. 13%), though roughly 6 months later both groups had improved (15% vs. 9%). Without data from a longer-term panel study, we cannot reconcile these divergent findings in a way that is definitive. However, one scenario that would be consistent with the results of all studies is that Jewish college students experienced acute declines in well-being following the October 7, 2023 attacks and resulting campus protests (as per the panel study), but had returned to their more typical levels of well-being by the time our survey launched (as per follow-up data in the panel study, our results, and historical patterns in HMS). It is also important to note that, notwithstanding differences in the timing of the survey rollouts, the results of these studies are not directly comparable. One involved Jewish students in German universities [24], whereas the other asked students to self-assess their mental health [25], which may elicit broader appraisals of one’s life compared to the more granular assessments of well-being dimensions in our survey.

Setting aside the uncertainty about trajectories and the comparability of studies, the results of our survey indicate that, on average, Jewish students were not experiencing lingering difficulties with mental health, social connection, or flourishing in early 2025. When these patterns are considered alongside the relatively prevalent concerns about antisemitism on campus, they suggest the Jewish students in our study were, as a group, resilient to what many perceived as a difficult campus atmosphere, and a more general trend of rising violence against Jews in American society [12]. This apparent resilience has been observed in research with other stigmatized and mistreated groups, suggesting there is considerable variability in how identity-based stressors, including concerns about a hostile atmosphere, relate to the well-being of group members [21,27,28,37].

Drawing on findings from the literature on identity-based stressors, we also considered whether the well-being of Jewish students would relate to their sense of belonging on campus and/or perceptions of institutional atmosphere toward Judaism and Israel. The results indicated that well-being was more strongly related to sense of belonging, which explained 2–4 times more variability in outcomes versus perceived atmosphere. Given that belonging was the stronger correlate of well-being, it is plausible that strong, supportive social connections buffered Jewish students from concerns about campus atmosphere. We did not administer the measures here necessary to test this hypothesis, but doing so should be a priority in future research. Given that a sense of belonging is associated with well-being for all demographic groups [5,38], interventions to foster belonging may be a promising policy lever for efforts to support college students’ well-being, relative to policies aimed at changing campus atmosphere. It is also plausible, of course, that the patterns observed here reflect adverse effects of social disconnection, as opposed to benefits of belonging. To settle this issue, studies could randomize Jewish students to a belonging intervention and measure effects on well-being [5].

We hope that our findings are not misinterpreted to suggest that universities have no reason to invest in efforts to safeguard Jewish students’ civil rights. The fact that Jewish students, on average, report comparatively similar well-being to other religiously identified peers, despite concerns about antisemitism on campus, does not absolve universities of the legal obligation to address discrimination. In addition to legal and civil rights obligations, research on other populations suggests reasons for university policy to address hostile climates, even when survey data indicates general well-being. Specifically, research has shown that even when Black youth report good mental health and success in school, they have worse cardiometabolic health [39]. These findings suggest there is a physiologic cost to resilience, where maintaining well-being in a hostile atmosphere undermines physical health. Future research could include biomarkers as a complement to survey data – not only to provide a more holistic picture of well-being but also to test for health effects that students themselves may not recognize or choose to disclose. Such work could inform campus initiatives as well as clinical and public health strategies aimed at promoting the long-term health of members of minority groups who, despite exposure to considerable identity-related stress, nevertheless find ways to thrive.

Several aspects of our research design limit the extent to which the findings can be generalized. First, as noted, the findings on Jewish student well-being must be interpreted in light of the timing of the survey, which launched roughly 8 months after the peak of the campus protests and 15 months after the attacks of October 7, 2023. Second, neither our sample nor HMS is demographically representative of college students nationally, nor of the political and religious diversity among Jewish college students (which we did not measure). In particular, the students we sampled tended to be strongly identified with Judaism and Zionism. As such, our sample over-represents the population of Jewish college students one would expect to be most negatively affected by anti-Jewish and/or anti-Israel hostility. If that is the case, then our estimates of Jewish student well-being are more likely to be conservative than not, and the average well-being of the broader Jewish student college population in early 2025 was, if anything, better than our findings on their own would suggest. Third, we categorized students as Jewish based on their responses to questions about religious identity (2025 survey) and affiliation (HMS). As a result, there may have been students who viewed themselves as culturally or ethnically Jewish, but religiously identified as agnostic or atheist and therefore were not categorized as Jewish in analyses. Fourth, the campus demonstrations in spring 2024 led to marked policy changes at many US colleges and to interventions by the federal government that included lawsuits, funding freezes, and investigations. This unique policy context makes it unlikely our results would generalize to Jewish college students residing in other nations.

Other features of the study design limit our ability to draw strong inferences from the results. First, all the data in the 2025 survey are cross-sectional in nature, precluding inferences about cause-and-effect relationships between students’ sense of belonging and perceptions of campus atmosphere, on the one hand, and student well-being, on the other. However, evidence from randomized trials of interventions to improve college student belonging gives credence to the hypothesis that greater belonging can positively influence well-being [5]. Second, as noted above, significant changes in government and university policy occurred in the months preceding our survey launch. When President Trump took office in January, 2025, even more dramatic policy changes began to unfold, many of which coincided with the administration of our survey. As such, we cannot determine how much the apparent resilience of Jewish students in our sample reflects the consequence of those policy changes versus a return to baseline levels of functioning. Finally, there were variations in the wording and format of the religion and demographic items between our survey and HMS. Those variations may have contributed to the slightly different patterns of results the studies observed in Jewish versus Christian students.

Despite these limitations, the study’s results indicate that following a period of campus turmoil, Jewish students on average had comparatively high levels of well-being. From a clinical perspective, this pattern of resilience is reassuring – as a group these students report good mental health, strong social connections, meaning in life, success in relationships, and optimism about the future. As discussed above, these findings mirror research with other devalued groups, and converge with the view that adversity does not inevitably undermine well-being. The findings on contributors to well-being indicate that sense of belonging is a stronger correlate than perceived campus atmosphere. If substantiated in future studies with stronger designs, these results would have direct policy implications for universities – specifically, that if they plan to deploy resources to bolster the well-being of student groups experiencing campus turmoil, belonging interventions are likely to yield greater benefits than efforts to change the campus atmosphere.

## Supporting information

S1 Table Well-being comparisons between Jewish students and those of other religions in 2025 survey, adjusted for neuroticism. (DOCX)

S2 Table Well-being comparisons between Jewish students and those of other religions in 2025 survey, adjusted for institutional selectivity. (DOCX)

S3 Table Well-being comparisons between Jewish students and those of other religions in 2025 survey, using Generalized Estimating Equations. (DOCX)

S4 Table Characteristics of participants in Healthy Minds Study, surveyed between 2016 and 2022. (DOCX)

S5 Table Results of linear regression analyses relating perceptions of campus atmosphere to well-being of Jewish students in 2025 survey (n = 2006). (DOCX)

S6 Table Results of linear regression analyses relating sense of belonging to well-being of Jewish students in 2025 survey (n = 2006). (DOCX)

S7 Table Results of linear regression analyses simultaneously relating perceptions of campus atmosphere and sense of belonging to well-being of Jewish students in 2025 survey (n = 2006). (DOCX)

S1 Fig S1a. Geographic distribution of 2025 survey participants. Created in R with the urbnmapr package, using US Census Bureau shapefiles. S1b. Geographic distribution of US Jewish population. Created in R with the urbnmapr package, using US Census Bureau shapefiles.
(TIFF)

S2 Fig Well-being by religious identity and survey year in HMS. (TIFF)

S3 Fig Prevalence of moderate/severe distress by religious identity and survey year in HMS. (TIFF)

S4 Fig Perceptions of campus atmosphere towards Judaism and Israel. (TIFF)

S1 Data Two2025 survey dataset. (XLSX)
