# Supplementary material for: The well-being of Jewish college students in the United States
Source: PLoS One. 2026 Sep 11;21(9):e0357478. doi: 10.1371/journal.pone.0357478 (PMC13567768; doi:10.1371/journal.pone.0357478)
Supplement: Supplementary file 1 [file pone.0357478.s001.docx]

**Table S1**. **Well-being comparisons between Jewish students and those of other religions in 2025 survey, adjusted for neuroticism.**

|  | **Christian** **-** **Jewish**  *Contrast Estimate (95% CI)* | **Other Religion - Jewish**  *Contrast Estimate (95% CI)* | **No Religion - Jewish**  *Contrast Estimate (95% CI)* |
| --- | --- | --- | --- |
| PHQ-8 Depression  (possible range 0-24) | 0.88 (0.48, 1.28) ***** | 0.76 (0.09, 1.42) * | 1.39 (0.91, 1.86) ***** |
| GAD-7 Anxiety  (possible range 0-21) | 0.49 (0.10, 0.87) * | 0.24 (-0.40, 0.88) | 0.65 (0.20, 1.10) ***** |
| Loneliness  (possible range 3-9) | 0.08 (-0.07, 0.22) | 0.05 (-0.19, 0.29) | 0.27 (0.10, 0.44) ***** |
| Flourishing  (possible range 8-64) | 0.09 (-0.44, 0.61) | 0.01 (-0.88, 0.88) | -1.60 (-2.23, -0.97) ***** |

***Notes***. Contrast estimates and confidence intervals are derived from analyses of variance, and are adjusted for age, gender, racial/ethnic identity, field of study, and neuroticism. Positive estimates indicate the comparison group has higher values on the well-being indicator relative to Jewish students; negative estimates indicate the opposite. * denotes 95% confidence interval that excludes the null value of 0.
