# Supplementary material for: The well-being of Jewish college students in the United States
Source: PLoS One. 2026 Sep 11;21(9):e0357478. doi: 10.1371/journal.pone.0357478 (PMC13567768; doi:10.1371/journal.pone.0357478)
Supplement: Supplementary file 2 [file pone.0357478.s002.docx]

**Table S2. Well-being comparisons between Jewish students and those of other religions in 2025 survey, adjusted for institutional selectivity.**

|  | **Christian** **-** **Jewish**  *Contrast Estimate (95% CI)* | **Other Religion - Jewish**  *Contrast Estimate (95% CI)* | **No Religion - Jewish**  *Contrast Estimate (95% CI)* |
| --- | --- | --- | --- |
| PHQ-8 Depression  (possible range 0-24) | 0.84 (0.38, 1.29) ***** | 0.55 (-0.19, 1.30) | 1.42 (0.89, 1.95) ***** |
| GAD-7 Anxiety  (possible range 0-21) | 0.35 (-0.11, 0.81) | -0.06 (-0.82, 0.70) | 0.68 (0.14, 1.22) ***** |
| Loneliness  (possible range 3-9) | 0.04 (-0.12, 0.20) | -0.01 (-0.27, 0.25) | 0.28 (0.09, 0.46) ***** |
| Flourishing  (possible range 8-64) | 0.07 (-0.52, 0.65) | -0.01 (-0.97, 0.96) | -1.95 (-2.63, -1.27) ***** |

***Notes***. Contrast estimates and confidence intervals are derived from analyses of variance, and are adjusted for age, gender, racial/ethnic identity, field of study, and institutional selectivity, as reflected in mean campus SAT score. Positive estimates indicate the comparison group has higher values on the well-being indicator relative to Jewish students; negative estimates indicate the opposite. * denotes 95% confidence interval that excludes the null value of 0.
