# Supplementary material for: The well-being of Jewish college students in the United States
Source: PLoS One. 2026 Sep 11;21(9):e0357478. doi: 10.1371/journal.pone.0357478 (PMC13567768; doi:10.1371/journal.pone.0357478)
Supplement: Supplementary file 3 [file pone.0357478.s003.docx]

**Table S3. Well-being comparisons between Jewish students and those of other religions in 2025 survey, using Generalized Estimating Equations.**

|  | **Christian** **-** **Jewish**  *Contrast Estimate (95% CI)* | **Other Religion - Jewish**  *Contrast Estimate (95% CI)* | **No Religion - Jewish**  *Contrast Estimate (95% CI)* |
| --- | --- | --- | --- |
| PHQ-8 Depression  (possible range 0-24) | 0.70 (0.14, 1.25) ***** | 0.53 (-0.15, 1.21) | 1.37 (0.78, 1.96) ***** |
| GAD-7 Anxiety  (possible range 0-21) | 0.32 (-0.19, 0.83) | -0.08 (-0.93, 0.77) | 0.73 (0.18, 1.28) ***** |
| Loneliness  (possible range 3-9) | 0.03 (-0.14, 0.20) | -0.03 (-0.32, 0.27) | 0.27 (0.05, 0.48) ***** |
| Flourishing  (possible range 8-64) | 0.31 (-0.32, 0.93) | 0.34 (-0.55, 1.23) | -1.57 (-2.36, -0.78) ***** |

**Notes**. Contrast estimates and confidence intervals are derived from Generalized Estimating Equations, specifying students’ college or university as a nesting factor and an exchangeable covariance matrix. Models are adjusted for age, gender, racial/ethnic identity, and field of study. Positive values indicate the comparison group has higher scores on indicator relative to Jewish students; negative values indicate the opposite. * indicates 95% confidence interval excludes the null value of 0.
