# Supplementary material for: The well-being of Jewish college students in the United States
Source: PLoS One. 2026 Sep 11;21(9):e0357478. doi: 10.1371/journal.pone.0357478 (PMC13567768; doi:10.1371/journal.pone.0357478)
Supplement: Supplementary file 4 [file pone.0357478.s004.docx]

**Table S4. Characteristics of participants in Healthy Minds Study, surveyed between 2016 and 2022.**

|  | **Full Sample**  (n=507,010) | **Jewish Students**  (n=10,904) | **Christian Students**  (n=172,879) | **Other Religion** (n=44,302) | **No Religion**  (n=143,090) |
| --- | --- | --- | --- | --- | --- |
|  | Mean (SD) or N (%) | Mean (SD) or N (%) | Mean (SD) or N (%) | Mean (SD) or N (%) | Mean (SD) or N (%) |
| Age, years | 21.8 (5.8) | 20.7 (4.0) | 21.7 (5.8) | 22.5 (6.7) | 21.6 (5.0) |
| Identifies as man | 142,275 (28%) | 3,362 (31%) | 47,283 (27%) | 11,871 (27%) | 43,404 (30%) |
| Identifies as woman | 346,188 (68%) | 6,898 (63%) | 123,798 (72%) | 30,206 (68%) | 92,435 (65%) |
| Identifies as White | 314,641 (62%) | 8,926 (82%) | 113,844 (66%) | 16,969 (38%) | 94,945 (66%) |
| Identifies as Asian | 46,498 (9%) | 115 (1%) | 9,122 (5%) | 11,448 (26%) | 14,609 (10%) |
| Identifies as Hispanic | 42,299 (8%) | 177 (2%) | 18,096 (10%) | 1,826 (4%) | 8,677 (6%) |
| Identifies as Middle Eastern | 5,674 (1%) | 123 (1%) | 536 (0.3%) | 2,838 (6%) | 706 (0.5%) |
| Identifies as Black | 37,857 (7%) | 64 (0.6%) | 13,907 (8%) | 4,952 (10%) | 6,573 (5%) |
| Major - STEM field | 134,054 (26%) | 2,191 (20%) | 46,981 (27%) | 11,946 (27%) | 38,824 (27%) |
| Major - Social sciences | 140,271 (28%) | 2,582 (24%) | 53,182 (31%) | 11,333 (26%) | 34,672 (24%) |
| Major - Humanities | 54,611 (11%) | 1,630 (15%) | 15,118 (9%) | 5,054 (11%) | 20,713 (14%) |

***Note***. Values for racial/ethnic identity exceed 100%, because students can endorse multiple identities. On all characteristics, Jewish students differ from at least one other group at *p* < .001.
