# Supplementary material for: The well-being of Jewish college students in the United States
Source: PLoS One. 2026 Sep 11;21(9):e0357478. doi: 10.1371/journal.pone.0357478 (PMC13567768; doi:10.1371/journal.pone.0357478)
Supplement: Supplementary file 5 [file pone.0357478.s005.docx]

**Table S5. Results of linear regression analyses relating perceptions of campus atmosphere to well-being of Jewish students in 2025 survey (n=2006).**

|  | **Depression**  *B (95% CI)* | **Anxiety**  *B (95% CI)* | **Loneliness**  *B (95% CI)* | **Flourishing**  *B (95% CI)* |
| --- | --- | --- | --- | --- |
| Age (years) | -0.03 (-.11, .05) | 0.02 (-.06, .10) | 0.01 (-.02, .04) | 0.00 (-.10, .11) |
| Gender identity (woman) | 0.05 (-.34, .43) | 1.11 (.71, 1.52) * | -0.13 (-.27, .00) | 1.55 (1.04, 2.05) |
| Racial/ethnic identity (Black) | 2.36 (.67, 4.05) * | 2.54 (.77, 4.30) * | 0.89 (.29, 1.50) * | -4.20 (-6.43, -1.97) * |
| Racial/ethnic identity (Asian) | 0.48 (-.78, 1.74) | 0.43 (-.88, 1.75) | 0.07 (-.38, .52) | -0.14 (-1.80, 1.52) |
| Racial/ethnic identity (Hispanic) | 0.14 (-.57, .84) | 0.05 (-.68, .79) | -0.19 (-.44, .07) | 0.87 (-.06, 1.80) |
| Racial/ethnic identity (Middle Eastern) | 0.75 (.15, 1.36) * | 0.35 (-.29,.98) | 0.13 (-.09, .35) | -0.04 (-.84, .76) |
| Racial/ethnic identity (Other) | 0.74 (-.10, 1.58) | 0.77 (-.11, 1.64) | 0.70 (.40, 1.00) * | -0.18 (-1.29, .93) |
| Academic focus (STEM) | -0.05 (-.48, .37) | -0.23 (-.67, .22) | 0.04 (-.11, .19) | -0.06 (-.62, .51) |
| Academic focus (Social science) | -0.17 (-.62, .28) | -0.20 (-.67, .26) | 0.06 (-.10, .22) | 0.09 (-.56, .68) |
| Campus atmosphere (SD units) | 0.56 (.37, .75) * | 0.81 (.61, 1.01) * | 0.26 (.19, .33) * | -0.66 (-.91, -.41) * |
| Total variance explained (R^2^) | .17 | .24 | .23 | .20 |
| Unique variance explained by campus atmosphere (ΔR^2^) | .06 | .08 | .07 | .04 |

***Note***. Racial/ethnic identity is modeled such that students who identify as White are the reference category. Academic focus is modeled such that students majoring in Humanities are the reference category. Higher scores on the campus atmosphere measure indicate students perceive more hostility towards Israel and Judaism.
