# Supplementary material for: The well-being of Jewish college students in the United States
Source: PLoS One. 2026 Sep 11;21(9):e0357478. doi: 10.1371/journal.pone.0357478 (PMC13567768; doi:10.1371/journal.pone.0357478)
Supplement: Supplementary file 6 [file pone.0357478.s006.docx]

**Table S6. Results of linear regression analyses relating sense of belonging to well-being of Jewish students in 2025 survey (n=2006)**

|  | **Depression**  *B (95% CI)* | **Anxiety**  *B (95% CI)* | **Loneliness**  *B (95% CI)* | **Flourishing**  *B (95% CI)* |
| --- | --- | --- | --- | --- |
| Age (years) | -0.08 (-.16, -.01) * | -0.03 (-.10, .05) | -0.01 (-.04, .02) | 0.09 (-.01, .18) |
| Gender identity (woman) | 0.31 (-.06, .67) | 1.41 (1.02, 1.80) * | -0.02 (-.15, .11) | 1.16 (.69, 1.63) * |
| Racial/ethnic identity (Black) | 1.85 (.25, 3.46) * | 2.04 (.34, 3.74) * | 0.68 (.12, 1.24) * | -3.38 (-5.43, -1.33) * |
| Racial/ethnic identity (Asian) | 0.47 (-.72, 1.66) | 0.48 (-.78, 1.74) | 0.07 (-.34, .49) | -0.07 (-1.60, 1.45) |
| Racial/ethnic identity (Hispanic) | 0.06 (-.61, .73) | -0.03 (-.74, .67) | -0.22 (-.45, .01) | 0.98 (.13, 1.84) * |
| Racial/ethnic identity (Middle Eastern) | 0.42 (-.15, .99) | 0.12 (-.49, .72) | 0.00 (-.20, .20) | 0.59 (-.15, 1.32) |
| Racial/ethnic identity (Other) | 0.17 (-.63, .97) | 0.30 (-.55, 1.14) | 0.47 (.19, .75) * | 0.82 (-.20, 1.85) |
| Academic focus (STEM) | 0.02 (-.39, .42) | -0.18 (-.60, .25) | 0.06 (-.08, .21) | -0.17 (-.69, .34) |
| Academic focus (Social science) | 0.00 (-.42, .43) | -0.04 (-.49, .41) | 0.13 (-.02, .28) | -0.20 (-.74, .35) |
| Sense of belonging (SD units) | -1.50 (-1.68, -1.32) * | -1.51 (-1.70, -1.32) * | -0.63 (-.69, -.57) * | 2.38 (2.15, 2.61) * |
| Total variance explained (R^2^) | .36 | .36 | .43 | .44 |
| Unique variance explained by  sense of belonging (ΔR^2^) | .12 | .10 | .16 | .16 |

***Note***. Racial/ethnic identity is modeled such that students who identify as White are the reference category. Academic focus is modeled such that students majoring in Humanities are the reference category. Higher scores on the sense of belonging measure indicate students feel a greater sense of belonging and connection to campus.
