# Supplementary material for: The well-being of Jewish college students in the United States
Source: PLoS One. 2026 Sep 11;21(9):e0357478. doi: 10.1371/journal.pone.0357478 (PMC13567768; doi:10.1371/journal.pone.0357478)
Supplement: Supplementary file 7 [file pone.0357478.s007.docx]

**Table S7. Results of linear regression analyses simultaneously relating perceptions of campus atmosphere and sense of belonging to well-being of Jewish students in 2025 survey (n=2006)**

|  | **Depression**  *B (95% CI)* | **Anxiety**  *B (95% CI)* | **Loneliness**  *B (95% CI)* | **Flourishing**  *B (95% CI)* |
| --- | --- | --- | --- | --- |
| Age (years) | -0.08 (-.16, -.01) * | -0.03 (-.11, .05) | -0.01 (-.04, .02) | 0.08 (-.01, .18) |
| Gender identity (woman) | 0.30 (-.07, .67) | 1.35 (.97, 1.74) * | -0.03 (-.16, .10) | 1.12 (.65, 1.60) * |
| Racial/ethnic identity (Black) | 1.86 (.26, 3.46) * | 2.06 (.37, 3.76) * | 0.68 (.12, 1.25) * | -3.37 (-5.42, -1.32) * |
| Racial/ethnic identity (Asian) | 0.46 (-.73, 1.66) | 0.42 (-.84, 1.68) | 0.07 (-.35, .48) | -0.11 (-1.64, 1.41) |
| Racial/ethnic identity (Hispanic) | 0.06 (-.60, .73) | -0.02 (-.73, .69) | -0.22 (-.45, .02) | 0.99 (.14, 1.85) * |
| Racial/ethnic identity (Middle Eastern) | 0.41 (-.16, .99) | 0.03 (-.58, .64) | -0.01 (-.21, .19) | 0.52 (-.21, 1.26) |
| Racial/ethnic identity (Other) | 0.16 (-.64, .96) | 0.23 (-.62, 1.07) | 0.46 (.18, .74) * | 0.78 (-.25, 1.80) |
| Academic focus (STEM) | 0.02 (-.39, .42) | -0.17 (-.59, .26) | 0.07 (-.08, .21) | -0.17 (-.68, .35) |
| Academic focus (Social science) | 0.00 (-.42, .43) | -0.04 (-.49, .41) | 0.13 (-.02, .28) | -0.20 (-.74, .35) |
| Sense of belonging (SD units) | -1.49 (-1.68, -1.29) * | -1.39 (-1.60, -1.19) * | -0.62 (-.68, -.55) * | 2.54 (2.21, 2.70) * |
| Campus atmosphere (SD units) | .04 (-.15, .23) | .32 (.12, .52) * | 0.04 (-.03, .11) | 0.21 (-.03, .46) |

***Note***. Racial/ethnic identity is modeled such that students who identify as White are the reference category. Academic focus is modeled such that students majoring in Humanities are the reference category.
