# Supplementary material for: The well-being of Jewish college students in the United States
Source: PLoS One. 2026 Sep 11;21(9):e0357478. doi: 10.1371/journal.pone.0357478 (PMC13567768; doi:10.1371/journal.pone.0357478)
Supplement: Supplementary file 8 [file pone.0357478.s014.docx]

August 5, 2026

Dr. Vasileios Kallinterakis
Academic Editor - *PLOS One*

Dear Dr. Kallinterakis -

Thanks for the prompt review of our manuscript “*The Well-Being of Jewish College Students in the United States* (PONE-D-26-20428).” Drawing on the helpful recommendations from reviewers, we revised the paper extensively, and believe it has improved considerably as a result. In the letter below, we respond point-by-point to your comments and those of the reviewers.

**Editor’s Comments**

Comment 1 - Style

Please ensure that your manuscript meets PLOS ONE's style requirements.

Response 1 - Style

We reviewed the style templates and revised the submission accordingly.

Comment 2 - Code

Please review our guidelines ... and ensure that your code is shared in a way that follows best practice and facilitates reproducibility and reuse.

Response 2 - Code

We read the guidelines carefully, and don’t believe that in our paper “*author-generated code underpins the findings*.” Of course, we could be mis-interpreting the guidelines. If there’s specific code you have in mind - e.g., syntax used for statistical analysis - we’re happy to post it on OSF along with the pre-registration and survey materials.

Comment 3 - Acknowledgments

Please remove any funding-related text from the manuscript and let us know how you would like to update your Funding Statement.

Response 3 - Acknowledgments

Sorry for the mistakes. We removed the Acknowledgement section from the manuscript, since there’s no relevant material to place there. And here’s an updated funding statement, which contains all the information you requested.

“This research was supported by grants from an Anonymous foundation (to GEM, #2024-5981)

and Northwestern University (to SIL, #10029690). The funders had no role in the design, analysis, or writing of study.”

Comment 4 - Funding Statement

We note that the grant information you provided in the ‘Funding Information’ and ‘Financial Disclosure’ sections do not match.

Response 4 - Funding Statement

Again, sorry about the mistake - see response above for an updated funding statement. We have added this material to the Funding Disclosure section in the manuscript portal.

Comment 5 - Supporting Info

Please include captions for your Supporting Information files at the end of your manuscript, and update any in-text citations to match accordingly.

Response 5 - Supporting Info

We updated the manuscript as directed, see p. 33.

Comment 6 - Citations

If the reviewer comments include a recommendation to cite specific previously published works, please review and evaluate these publications to determine whether they are relevant and should be cited. There is no requirement to cite these works unless the editor has indicated otherwise. 

Response 6 - Citations

Thanks for the clarification on this issue.

**Reviewer #1**

Comment 1 - Abstract

-Line 31 “To fill these gaps in knowledge.” remove period which appears mid sentence.

-Lines 31-32 Unclear how student well-being one year later (January-June 2025) exactly relates to student well-being a year earlier when Israel-Hamas protests/conflict-related incidents were at their peak (Spring 2024). Geopolitical events and climate shift rapidly- a year later, the climate may not be ideal for measuring how students experienced the aforementioned events. There needs to be a bit more rationale here as to the time lag. Anti-semitism is ongoing- but the authors mention the events of October 7 and clearly highlight spring 2024. Yet the data timeline does not align with that.

-Line 35 Second aim is lengthy/reads unwieldy. Perhaps streamline to support the readability.

-Specific survey content including tools used not specified in Abstract, limiting the reader’s understanding of the scope of data collection. This can be added in succinctly/briefly for clarification.

Response 1 - Abstract

Thanks. We’ve incorporated these helpful suggestions into the revised abstract.

Comment 2 - Introduction

-Lines 64-65 “ Nearly one-third” - report or reported? The rest of this paragraph is past tense.

-Lines 67-70 Sentence structure does not make sense. Please check.

-Lines 73-74 Needs its’ own citations - UCLA task force is not (alone) ‘policymakers and politicians’ as stated.

-Lines 79-83 It would be helpful to foreshadow some of the mentioned ‘range of protective factors’ as these are relevant later on in the paper.

-Lines 84-94 This paragraph is clear and timely- however racially minoritized youth is not mentioned previously- and this bears mention since Jewish students are a sub-group of this- and previous research does exist within other racially minoritized student groups- relevant to this study.

Response 2 - Introduction

Again, thanks for the helpful feedback. We have extensively revised the Introduction, incorporating these suggestions along with recommendations from the other reviewer.

Comment 3 - Methods and Materials

- Lines 135-149 Were these questions asked only of Jewish students?

- Line 151-159 Health Minds Study requires citation

- Line 175 (179) How was neuroticism assessed? You mention a 3 item scale, but the flow of this section is disjointed. I suggest a clear flow where the entire survey content, who receive which questions etc. is included, rather than a narrative which seems to switch back and forth between survey content and data analysis. (You may include this in a chart/table for clarity.)

Response 3 - Methods and Materials

Making these changes has improved the clarity of the section. At the outset of the measures section, we now provide a hyperlink to the full text of the survey (line 148) and state that unless otherwise specified, all instruments were administered to all participants (lines 148-150). We also provide citations for papers and manuals relevant to the Healthy Minds Study (line 192) and clarify how neuroticism was measured (lines 219-220).

Comment 4 - Positionality Statement

No positionality statements are included. Given the subject matter of this manuscript, strongly suggest to include. Topics such as Jewish identity, belonging, campus climate and Israel-Hamas conflict are politically and emotionally charged. Why are the authors uniquely qualified to study this, not only from a professional perspective but also from a personal one? If not uniquely qualified, please provide some context as to background/potential bias. This information provides context.

Response 4 - Positionality Statement

Although all of the authors of the study identify as Jewish and are concerned about rising antisemitism in the US,  we have chosen not to include a positionality statement, as we believe it is not warranted for the current study. In qualitative data collection, positionality statements can be helpful, because (for instance), in conducting interviews and field observations, the researcher is the instrument of data collection. Data are produced through interactions involving the researcher herself. Moreover, the interpretive and iterative nature of qualitative data analysis means that a researcher’s personal biography and concerns can shape which findings she chooses to make central and which are more peripheral.  The current study, by contrast, uses standardized survey instruments. Moreover, the surveys were not administered by any particular individual; participants responded to survey questions on an online platform. Finally, we preregistered our hypotheses and analytic procedures to guard against potential biases and dispositions that might shape our interpretation of the results.

Comment 5 - Results

Line 270 “Those who are Jewish” rather state “Those who identified as Jewish”

Response 5 - Results

We have updated this language. Also, given the other reviewer’s comments regarding this issue, we have explained in multiple places that our categorization is based on students’ religious identity.

Comment 6 - Discussion

Discussion needs to be situated in more literature (and add this to introduction as well). Also, can split up with/without subheadings into Discussion, Limitations, Implications, Conclusion. Also, please organize the content to flow with longer, more coherent paragraphs.

Response 6 - Discussion

Thanks for these helpful suggestions. We substantially revised the content and structure of the Discussion, guided by these suggestions and those from the other reviewer.

**Reviewer #2**

Comment 1 - General

Thank you for this interesting and timely study examining Jewish student well-being in the post-October 7th campus climate. The topic is important and the data have the potential to make a meaningful contribution to the literature. However, I have identified several methodological and framing concerns that I believe must be addressed to strengthen the paper for publication.

Response 1 - General

Thanks for the kind words about the paper.

Comment 2 - Preregistration

The preregistration was not available on OSF—only the survey was uploaded. Please make the preregistration publicly available so that adherence to the preregistered analytic plan can be evaluated.

Response 2 - Preregistration

We apologize for this mistake. The revised manuscript provides the correct hyperlink to the preregistration materials (line 129).

Comment 3 - Introduction

The introduction would benefit from a brief description of the events of October 7th (i.e., the terrorist attack and ensuing war) to contextualize the study for readers unfamiliar with the timeline. Consider noting that upticks in antisemitism are often reported globally following Israeli military action, as this provides important historical context. Relatedly, the statement "encampments that centered Zionism and the Israeli government" is vague and could be elaborated to more fully capture the nuance of the encampment formation.

The introduction should also discuss how the US government response differed from that of other countries—where protests and encampments were largely permitted to continue—making the US a unique case study.

The study's framing would benefit from significant revision to reflect the timeline of data collection with reference to sociopolitical events. Your data capture Jewish student well-being after substantial governmental and institutional attention had been given to the upsurge in antisemitism on college campuses. As such, the paper cannot be characterized as an examination of heightened antisemitism on college campuses without the important contextual detail that institutions had, often as a result of direct or indirect action from the government, implemented policies or procedures in an attempt to address Jewish students' civil rights. This temporal and policy context may explain why findings diverge from other studies conducted during this period and should be foregrounded throughout.

Please include specific hypotheses in the introduction, including hypotheses for the HMS data, whose inclusion is otherwise unclear.

Response 3 - Introduction

Thanks for the helpful suggestions. We’ve incorporated them - along with suggestions from Reviewer 1 - into the revision. As a result of these changes, the Introduction has improved considerably. The only recommendation we’ve passed on is the one to include specific hypotheses. As the pre-registration materials indicate, we did not begin the study with “*strong a priori hypothesis about the well-being of Jewish students relative to their peers of other religious identities. ... our goal (was) not to test a specific hypothesis, but instead to accurately describe the well-being of Jewish students, by accruing a large, diverse sample...*” With that said, we have added material to the Introduction clarifying the rationale for HMS analyses (lines 114-118). Finally, the point about the US as a “unique case study” is an important one. We now raise it in the section of the Discussion about generalizability, where it ties in with other concerns about what our sample can - and can’t - tell readers about the well-being of Jewish students (lines 455-458).

Comment 4: Measures & Survey Design

Judaism is framed as a religious identity, which is a notable limitation. Most recent studies (e.g., your reference #26) avoid this framing. Many Jews identify as atheist or agnostic, and this group may be particularly affected by campus climate through lower levels of connection to the Jewish community via institutionalized religious practice. Please make explicit throughout the paper that you are assessing Judaism in the religious sense and not as an ethnicity, and discuss how this framing may have impacted your data and results.

Response 4: Measures & Survey Design

This is an important point. We highlight it in the Methods (lines 156-159), where the approach to measuring religion is introduced, and again In the Discussion, where we consider how it may have contributed to the results (lines 451-455).

Comment 5 - Other Responses

Most demographic items appear to have included an open-ended response option in the survey uploaded to OSF. Was this also the case for religion? If not, please explain the rationale. If so, elaborate on how "other" responses were coded.

Response 5 - Other Responses

Unlike the other demographic items, the question about religion item did not provide an option for an open-ended response. This was a clerical error on our part. However, this item did have the response option “Other religion not specified.” As we now state on line 162, students who selected this option were placed in the “Other Religion” category.

Comment 6 - Jewish ethnicity

For the race/ethnicity item, did any respondents identify as Jewish (or Ashkenazi, Sephardic, Mizrahi)? If so, how were these responses handled? This relates directly to the concern about respondents who identify as ethnically but not religiously Jewish.

Response 6 - Jewish ethnicity

No students responded in this way.

Comment 7 - Gender

Please clarify how gender was coded. The paper states that "female" was used as the reference category, but the survey uses the term "woman" and includes six other gender categories. Collapsing men with all other categories makes little theoretical sense. Please explain and justify the coding scheme.

Response 7 - Gender

These are good questions and we apologize for the confusion - our bad. We’ve updated the paper to clarify our rationale and approach to modeling gender (see lines 213-217). We opted to code gender into two categories (female vs. other) to harmonize our survey with results from HMS, which until the later years did not have more granular response options. To make sure this approach didn’t bias our results, we reconducted the primary analyses after excluding 89 students in our survey (2.6%) whose responses were not either “man” or “woman.” The findings were identical. Throughout the paper and tables, we have replaced “female” with “woman.” Thanks for catching this.

Comment 8 - HMS

Please describe how religious identity is assessed in the HMS study, and how HMS demographic covariates were assessed and may differ from those in the present study.

Response 8 - HMS

We now provide more detail about these items on lines 192-199. We also note in the Discussion that variations in the wording/format of these items may have contributed to the slightly different patterns of results observed in the two studies (lines 471-473).

Comment 9 - Campus atmosphere

Please elaborate on how the scale to capture campus atmosphere was created and how items were selected.

Response 9 - Campus atmosphere

We added material on these items to the Methods, see lines 177-187.

Comment 10 - Error describing item

There appears to be an error in the scale items listed at lines 144–147. I believe the first item should be "In order to fit in on my campus, I feel the need to hide that I am Jewish," with the remaining items following accordingly. Please verify and correct.

Response 10 - Error describing item

Thanks for catching this mistake. The section now reads as it should (lines 182-183).

Comment 11 - Misc suggestions

- Please include how neuroticism was assessed in the methods section.

- Cronbach's "a" should be "α" throughout.

- Line 172: "ethnic identify" should be "ethnic identity."

Response 12 - Misc suggestions

Thanks for catching these errors and omissions, we have corrected them.

Comment 13 - Analytic Approach

Please elaborate on why GEEs were used rather than multilevel modelling. I would suggest that fitting a multilevel model with students nested within institutions (or a three-level model: students within institutions within states if sample sizes permit) would be more informative. It would also be helpful to report intraclass correlation coefficients for target constructs.

Related to my comment above, the primary models reported should, by default, adequately account for the nested structure of the data. Please report these in the main text or provide justification for why adjustment is unnecessary (e.g., low ICCs).

Response 13 - Analytic Approach

We gave these suggestions a great deal of thought. In the end, we decided to keep the modeling strategy presented in the original paper, for three reasons we hope the reviewer can appreciate.

First, it matches the analytic strategy that we outlined in the pre-registration. (Which we realize was not available to the reviewer.) Second, there was little clustering of well-being within institutions - the intraclass correlation coefficients for the four measures were all < .03. We now report these results on lines 222-226. Finally, given the aims of this paper we believe GEE is a good approach. It manages the biases that can arise with nesting just as effectively as an MLM would. It doesn’t partition variance in the way an MLM does - so it won’t answer questions like “Does religious identity relate to well-being differently across institutions?” However, our study was not designed to answer questions like this and, even if doing so was of interest, the sample doesn’t have a sufficiently large numbers of respondents at multiple schools for that approach to yield meaningful results.

Comment 14 - Dummy coding

I found the dummy coding scheme unclear and believe this section could benefit from greater elaboration and explanation. For example, "reference" typically denotes the group coded as 0, but "referent" here appears to denote the included rather than omitted group. Additionally, the reference/base category for field of study is not specified. Please clarify.

Response 14 - Dummy coding

We updated this situation to clarify the coding scheme (see lines 213-217). The models should be much easier to interpret as a result. Thanks for this suggestion.

Comment 15 - Missing data

Please elaborate on how missing data were handled.

Response 15 - Missing data

We had virtually no missing data in the 2025 survey. Two respondents gave partial responses to well-being items, and we excluded them from analysis (now noted on lines 149-150). We took the same approach with HMS (noted on lines 200-201).

Comment 16 - Race/ethnicity

In the notes for Table 3, it appears that race and ethnicity were both included as covariates in the HMS analyses, but in the present study this was assessed as a single construct. Please address this discrepancy.

Response 16 - Race/ethnicity

We updated the paper and tables to indicate the approach to modeling racial/ethnic identity was identical across studies.

Comment 17 - Results & Interpretation Misc

- Avoid starting sentences with a numeral.

- Please be consistent in using either "%" or "percent" throughout.

- Claim that "these findings underscore an important point: identity-based stressors…do not have an automatic or inevitable relationship with well-being" is not substantiated by the data. The analyses are cross-sectional, and null findings cannot prove the absence of an effect

- The paragraph at lines 323–328 is written as though a within-person investigation was conducted.

Response 17 - Results & Interpretation Misc

These are all good points and we revised the manuscript to incorporate them.

Comment 18 - Buffering effect

You mention the buffering effect of social connection/support, and it appears this could be tested with your data. Including a supplementary analysis with an interaction term to test this buffering effect would strengthen the paper.

Response 18 - Buffering effect

We agree that an analysis of this nature would be valuable. However, the survey didn’t contain any questions that we believe adequately capture the construct of social buffering. The conventional approach to measuring this construct is to administer scales that inquire about the receipt and/or availability of emotional, tangible, and instrumental during stressful situations. Unfortunately, we did not have space in the protocol to include such measures. We have noted that future research should explicitly test this hypothesis and that we were not able to here (lines 415-416).

Comment 19 - Generalizability

Please make explicit in the discussion that results cannot be generalized beyond the US given the idiosyncratic nature of the US administration's response. Related to my comments regarding the introduction, the study could just as easily be framed as an examination of the efficacy of US administration attempts to combat antisemitism on college campuses. This contextual detail could be seen as both a strength and a limitation of your examination and should be central to how the results are interpreted.

Response 19 - Generalizability

We now discuss this issue on lines 455-458 and 465-470 of the revision.

Comment 20 - Policy changes

... By the time your study was fielded, such intervention efforts had already taken place. Your study could just as easily be framed as an indication of the success of these efforts. Please include discussion of this.

Response 20 - Policy changes

We agree with this important point - the policy changes made in response to campus turmoil could have contributed to the pattern of results we observed. We discuss this possibility on lines 465-470 of the revision.

Comment 21 - Citations

The paragraph beginning on line 373 includes several assertions that would benefit from citations. If this is speculation, please make this explicit. For example, it is just as reasonable to suspect that those with little connection to Jewish identity may be more distressed due to feelings of alienation.

Response 21 - Citations

Fair points. We further this developed this section by adding citations and mentioning the alternative interpretation the reviewer suggested (lines 419-422).

Comment 22 - HMS

Greater elaboration on the purpose of including the HMS data in your study would significantly strengthen the paper. Related to my comment about including specific hypotheses in the introduction, discussing how/if you expected results from your data to diverge from the HMS estimates would go a long way to address this.

Response 22 - HMS

See our response to Comment 3 above.

Comment 23 - Limitations

- The limitations section would benefit from significant expansion to address measurement limitations (particularly the religious framing of Jewish identity) and limited generalizability. Please also cite work conducted in the immediate aftermath of October 7th which does find that Jewish student well-being deteriorated in that period, when discussing how and why your findings might diverge.

- You mention that your sample was not representative of the broader population of Jewish students in terms of political diversity, but this variable was not assessed. The statement is therefore unfounded, though the omission of this variable is a significant limitation that warrants greater elaboration.

Response 23 - Limitations

We significantly revised and expanded the limitations section to reflect these points and others (see lines 439-473).

Comment 24 - Conclusion

Please include a concluding paragraph.

Response 24 - Conclusion

Included - see lines 474-486.

This concludes our responses to the referees’ comments. On behalf of the paper’s co-authors, I want to thank both of them for the helpful comments, and you for considering the manuscript at *PLOS One*. We look forward to your editorial decision.

Meanwhile, if you have any questions, please feel free to contact me by telephone or e-mail

Sincerely,

Gregory Miller, Ph.D.

Louis W. Menk Professor
