# Supplementary material for: The well-being of Jewish college students in the United States
Source: PLoS One. 2026 Sep 11;21(9):e0357478. doi: 10.1371/journal.pone.0357478 (PMC13567768; doi:10.1371/journal.pone.0357478)
Supplement: Peer review history

# Peer review: Decision Letter 0

**DOI:** 10.1371/journal.pone.0357478.r001

**Authors:** Vasileios Kallinterakis

16 Jun 2026

PONE-D-26-20428

The Well-Being of Jewish College Students in the United States

PLOS One

Dear Dr. Miller,

Thank you for submitting your manuscript to PLOS ONE. After careful consideration, we feel that it has merit but does not fully meet PLOS ONE’s publication criteria as it currently stands. Therefore, we invite you to submit a revised version of the manuscript that addresses the points raised during the review process.

The reviewers have identified a series of areas meriting improvement, primarily in terms of the paper's empirical design and discussion.

Please submit your revised manuscript by Aug 01 2026 11:59PM. If you will need more time than this to complete your revisions, please reply to this message or contact the journal office at plosone@plos.org. When you're ready to submit your revision, log on to https://www.editorialmanager.com/pone/ and select the 'Submissions Needing Revision' folder to locate your manuscript file.

Please include the following items when submitting your revised manuscript:

- A letter that responds to each point raised by the academic editor and reviewer(s). You should upload this letter as a separate file labeled 'Response to Reviewers'.
- A marked-up copy of your manuscript that highlights changes made to the original version. You should upload this as a separate file labeled 'Revised Manuscript with Track Changes'.
- An unmarked version of your revised paper without tracked changes. You should upload this as a separate file labeled 'Manuscript'.

If you would like to make changes to your financial disclosure, please include your updated statement in your cover letter. Guidelines for resubmitting your figure files are available below the reviewer comments at the end of this letter.

If applicable, we recommend that you deposit your laboratory protocols in protocols.io to enhance the reproducibility of your results. Protocols.io assigns your protocol its own identifier (DOI) so that it can be cited independently in the future. For instructions see: https://journals.plos.org/plosone/s/submission-guidelines#loc-laboratory-protocols. Additionally, PLOS ONE offers an option for publishing peer-reviewed Lab Protocol articles, which describe protocols hosted on protocols.io. Read more information on sharing protocols at https://plos.org/protocols?utm_medium=editorial-email&utm_source=authorletters&utm_campaign=protocols.

As the corresponding author, your ORCID iD is verified in the submission system and will appear in the published article. PLOS supports the use of ORCID, and we encourage all coauthors to register for an ORCID iD and use it as well. Please encourage your coauthors to verify their ORCID iD within the submission system before final acceptance, as unverified ORCID iDs will not appear in the published article. Only the individual author can complete the verification step; PLOS staff cannot verify ORCID iDs on behalf of authors.

We look forward to receiving your revised manuscript.

Kind regards,

Vasileios Kallinterakis

Academic Editor

PLOS One

Journal Requirements:

When submitting your revision, we need you to address these additional requirements.

1. Please ensure that your manuscript meets PLOS ONE's style requirements, including those for file naming. The PLOS ONE style templates can be found at

https://journals.plos.org/plosone/s/file?id=wjVg/PLOSOne_formatting_sample_main_body.pdf and

https://journals.plos.org/plosone/s/file?id=ba62/PLOSOne_formatting_sample_title_authors_affiliations.pdf

2. Please note that PLOS One has specific guidelines on code sharing for submissions in which author-generated code underpins the findings in the manuscript. In these cases, we expect all author-generated code to be made available without restrictions upon publication of the work. Please review our guidelines at https://journals.plos.org/plosone/s/materials-and-software-sharing#loc-sharing-code and ensure that your code is shared in a way that follows best practice and facilitates reproducibility and reuse.

3. Thank you for stating the following in the Acknowledgments Section of your manuscript:

“This research was supported by grants from an Anonymous foundation and Northwestern University. The funders had no role in design, analysis, or writing of study.”

We note that you have provided additional information within the Acknowledgements Section that is not currently declared in your Funding Statement. Please note that funding information should not appear in the Acknowledgments section or other areas of your manuscript. We will only publish funding information present in the Funding Statement section of the online submission form.

Please remove any funding-related text from the manuscript and let us know how you would like to update your Funding Statement. Currently, your Funding Statement reads as follows:

“This research was supported by grants from an Anonymous foundation (to GM) and Northwestern University (to SIL). The funders had no role in design, analysis, or writing of study.”

Please include your amended statements within your cover letter; we will change the online submission form on your behalf.

4. We note that the grant information you provided in the ‘Funding Information’ and ‘Financial Disclosure’ sections do not match.

When you resubmit, please ensure that you provide the correct grant numbers for the awards you received for your study in the ‘Funding Information’ section

5. Please include captions for your Supporting Information files at the end of your manuscript, and update any in-text citations to match accordingly. Please see our Supporting Information guidelines for more information: http://journals.plos.org/plosone/s/supporting-information.

6. If the reviewer comments include a recommendation to cite specific previously published works, please review and evaluate these publications to determine whether they are relevant and should be cited. There is no requirement to cite these works unless the editor has indicated otherwise.

Additional Editor Comments :

The reviewers have now reverted to me with their comments. Overall, they see merit in your paper, yet identify a series of issues warranting attention, primarily in terms of the empirical design and discussion of results. Admittedly, both reviewers have offered very detailed comments, and I strongly suggest you heed them while revising your paper.

[Note: HTML markup is below. Please do not edit.]

Reviewers' comments:

Reviewer's Responses to Questions

**Comments to the Author**

1. Is the manuscript technically sound, and do the data support the conclusions?

Reviewer #1: Yes

Reviewer #2: Partly

**********

2. Has the statistical analysis been performed appropriately and rigorously?

Reviewer #1: I Don't Know

Reviewer #2: Yes

**********

3. Have the authors made all data underlying the findings in their manuscript fully available?

The PLOS Data policy

Reviewer #1: No

Reviewer #2: No

**********

4. Is the manuscript presented in an intelligible fashion and written in standard English?

Reviewer #1: Yes

Reviewer #2: Yes

**********

Reviewer #1: Thank you for the opportunity to review this manuscript. Please see comments below delineated by section and line number.

Abstract

Line 31 “To fill these gaps in knowledge.” remove period which appears mid sentence.

Lines 31-32 Unclear how student well-being one year later (January-June 2025) exactly relates to student well-being a year earlier when Israel-Hamas protests/conflict-related incidents were at their peak (Spring 2024). Geopolitical events and climate shift rapidly- a year later, the climate may not be ideal for measuring how students experienced the aforementioned events. There needs to be a bit more rationale here as to the time lag. Anti-semitism is ongoing- but the authors mention the events of October 7 and clearly highlight spring 2024. Yet the data timeline does not align with that.

Line 35 Second aim is lengthy/reads unwieldy. Perhaps streamline to support the readability.

-Specific survey content including tools used not specified in Abstract, limiting the reader’s understanding of the scope of data collection. This can be added in succinctly/briefly for clarification.

Introduction

Lines 64-65 “ Nearly one-third” - report or reported? The rest of this paragraph is past tense.

Lines 67-70 Sentence structure does not make sense. Please check.

Lines 73-74 Needs its’ own citations - UCLA task force is not (alone) ‘policymakers and politicians’ as stated.

Lines 79-83 It would be helpful to foreshadow some of the mentioned ‘range of protective factors’ as these are relevant later on in the paper.

Lines 84-94 This paragraph is clear and timely- however racially minoritized youth is not mentioned previously- and this bears mention since Jewish students are a sub-group of this- and previous research does exist within other racially minoritized student groups- relevant to this study.

Methods and Materials

Lines 135-149 Please clarify- were these questions asked only of Jewish students? Were there other questions asked of the 3 other categories of students but not of Jewish students? In other words were campus atmosphere / belonging questions asked of all students or only Jewish? This is not clear in the current reading.

Line 151-159 Health Minds Study requires citation

Line 175 (179) How was neuroticism assessed? You mention a 3 item scale, but the flow of this section is disjointed. I suggest a clear flow where the entire survey content, who receive which questions etc. is included, rather than a narrative which seems to switch back and forth between survey content and data analysis. (You may include this in a chart/table for clarity.)

Strong suggestion: No positionality statements are included. Given the subject matter of this manuscript, strongly suggest to include. Topics such as Jewish identity, belonging, campus climate and Israel-Hamas conflict are politically and emotionally charged. Why are the authors uniquely qualified to study this, not only from a professional perspective but also from a personal one? If not uniquely qualified, please provide some context as to background/potential bias. This information provides context.

Results

Line 270 “Those who are Jewish” rather state “Those who identified as Jewish”

Discussion

Lines 334-336 This is an important point and requires more previous literature to situate this finding. (I stated this point as well in the Introduction comments.) In general, while little research may exist, there has been, since October 7, a growing body of research on this topic which unfortunately was not included here. The paper reads as a standalone island with very little history, when in fact, there are studies that pre-date this one on similar topics. Strong suggestion- include all relevant studies in the Introduction and again in the Discussion to better situate results within the literature.

Structure- Discussion reads as a series of mini-points, rather than paragraphs. If this aligns with the conventions of the journal, then it does not need revision. However, most papers include paragraphs with 4-8 sentences each, allowing a smoother flow for the reader.

Ex. Lines 342-346 paragraph reads as a limitation (that section is missing here too). It does not flow from the previous one or into the subsequent one.

Lines 347-351 suggestion to put this into a formal “Conclusions” section. It reads that way.

Lines 352-358 belongs earlier in the discussion chater.

Lines 359-365 Can be toward the end of Discussion or in ‘Future Research/Implications for Research’ section

Lines 366-372 Another limitations point. Why in its’ own paragraph and not a formal limitations section?

Lines 373-381, 382-383 see comment on 366-372.

Suggestion: Discussion needs to be situated in more literature (and add this to introduction as well). Also, can split up with/with out subheadings into Discussion, Limitations, Implications, Conclusion. Also, please organize the content to flow with longer, more coherent paragraphs.

Reviewer #2: Thank you for this interesting and timely study examining Jewish student well-being in the post-October 7th campus climate. The topic is important and the data have the potential to make a meaningful contribution to the literature. However, I have identified several methodological and framing concerns that I believe must be addressed to strengthen the paper for publication.

Preregistration

The preregistration was not available on OSF—only the survey was uploaded. Please make the preregistration publicly available so that adherence to the preregistered analytic plan can be evaluated.

Introduction

The introduction would benefit from a brief description of the events of October 7th (i.e., the terrorist attack and ensuing war) to contextualize the study for readers unfamiliar with the timeline. Consider noting that upticks in antisemitism are often reported globally following Israeli military action, as this provides important historical context. Relatedly, the statement "encampments that centered Zionism and the Israeli government" is vague and could be elaborated to more fully capture the nuance of the encampment formation.

The introduction should also discuss how the US government response differed from that of other countries—where protests and encampments were largely permitted to continue—making the US a unique case study.

The study's framing would benefit from significant revision to reflect the timeline of data collection with reference to sociopolitical events. Your data capture Jewish student well-being after substantial governmental and institutional attention had been given to the upsurge in antisemitism on college campuses. As such, the paper cannot be characterized as an examination of heightened antisemitism on college campuses without the important contextual detail that institutions had, often as a result of direct or indirect action from the government, implemented policies or procedures in an attempt to address Jewish students' civil rights. This temporal and policy context may explain why findings diverge from other studies conducted during this period and should be foregrounded throughout.

Please include specific hypotheses in the introduction, including hypotheses for the HMS data, whose inclusion is otherwise unclear.

Methods: Measures & Survey Design

Judaism is framed as a religious identity, which is a notable limitation. Most recent studies (e.g., your reference #26) avoid this framing. Many Jews identify as atheist or agnostic, and this group may be particularly affected by campus climate through lower levels of connection to the Jewish community via institutionalized religious practice. Please make explicit throughout the paper that you are assessing Judaism in the religious sense and not as an ethnicity, and discuss how this framing may have impacted your data and results.

Most demographic items appear to have included an open-ended response option in the survey uploaded to OSF. Was this also the case for religion? If not, please explain the rationale. If so, elaborate on how "other" responses were coded.

For the race/ethnicity item, did any respondents identify as Jewish (or Ashkenazi, Sephardic, Mizrahi)? If so, how were these responses handled? This relates directly to the concern about respondents who identify as ethnically but not religiously Jewish.

Please clarify how gender was coded. The paper states that "female" was used as the reference category, but the survey uses the term "woman" and includes six other gender categories. Collapsing men with all other categories makes little theoretical sense. Please explain and justify the coding scheme.

Please describe how religious identity is assessed in the HMS study, and how HMS demographic covariates were assessed and may differ from those in the present study.

Please elaborate on how the scale to capture campus atmosphere was created and how items were selected.

There appears to be an error in the scale items listed at lines 144–147. I believe the first item should be "In order to fit in on my campus, I feel the need to hide that I am Jewish," with the remaining items following accordingly. Please verify and correct.

Please include how neuroticism was assessed in the methods section.

Cronbach's "a" should be "α" throughout.

Line 172: "ethnic identify" should be "ethnic identity."

Methods: Analytic Approach

Please elaborate on why GEEs were used rather than multilevel modelling. I would suggest that fitting a multilevel model with students nested within institutions (or a three-level model: students within institutions within states if sample sizes permit) would be more informative. It would also be helpful to report intraclass correlation coefficients for target constructs.

Related to my comment above, the primary models reported should, by default, adequately account for the nested structure of the data. Please report these in the main text or provide justification for why adjustment is unnecessary (e.g., low ICCs).

I found the dummy coding scheme unclear and believe this section could benefit from greater elaboration and explanation. For example, "reference" typically denotes the group coded as 0, but "referent" here appears to denote the included rather than omitted group. Additionally, the reference/base category for field of study is not specified. Please clarify.

Please elaborate on how missing data were handled.

In the notes for Table 3, it appears that race and ethnicity were both included as covariates in the HMS analyses, but in the present study this was assessed as a single construct. Please address this discrepancy.

Results & Interpretation

Avoid starting sentences with a numeral.

Please be consistent in using either "%" or "percent" throughout.

The claim that "these findings underscore an important point: identity-based stressors…do not have an automatic or inevitable relationship with well-being" is not substantiated by the data. The analyses are cross-sectional, and null findings cannot prove the absence of an effect (absence of evidence is not evidence of absence). Please revise.

The paragraph at lines 323–328 is written as though a within-person investigation was conducted.

Discussion

You mention the buffering effect of social connection/support, and it appears this could be tested with your data. Including a supplementary analysis with an interaction term to test this buffering effect would strengthen the paper.

Please make explicit in the discussion that results cannot be generalized beyond the US given the idiosyncratic nature of the US administration's response. Related to my comments regarding the introduction, the study could just as easily be framed as an examination of the efficacy of US administration attempts to combat antisemitism on college campuses. This contextual detail could be seen as both a strength and a limitation of your examination and should be central to how the results are interpreted.

Related to my above comment, the statement "We hope that our findings are not misinterpreted to suggest that universities have no reason to invest in efforts to safeguard Jewish students' civil rights" should be followed by an acknowledgment that by the time your study was fielded, such intervention efforts had already taken place. Your study could just as easily be framed as an indication of the success of these efforts. Please include discussion of this.

The paragraph beginning on line 373 includes several assertions that would benefit from citations. If this is speculation, please make this explicit. For example, it is just as reasonable to suspect that those with little connection to Jewish identity may be more distressed due to feelings of alienation.

Greater elaboration on the purpose of including the HMS data in your study would significantly strengthen the paper. Related to my comment about including specific hypotheses in the introduction, discussing how/if you expected results from your data to diverge from the HMS estimates would go a long way to address this.

Limitations

The limitations section would benefit from significant expansion to address measurement limitations (particularly the religious framing of Jewish identity) and limited generalizability. Please also cite work conducted in the immediate aftermath of October 7th which does find that Jewish student well-being deteriorated in that period, when discussing how and why your findings might diverge.

You mention that your sample was not representative of the broader population of Jewish students in terms of political diversity, but this variable was not assessed. The statement is therefore unfounded, though the omission of this variable is a significant limitation that warrants greater elaboration.

Conclusion

Please include a concluding paragraph.

**********

what does this mean?). If published, this will include your full peer review and any attached files.

If you choose “no”, your identity will remain anonymous but your review may still be made public.

**Do you want your identity to be public for this peer review?**  For information about this choice, including consent withdrawal, please see our Privacy Policy

Reviewer #1: No

Reviewer #2: No

**********

[NOTE: If reviewer comments were submitted as an attachment file, they will be attached to this email and accessible via the submission site. Please log into your account, locate the manuscript record, and check for the action link "View Attachments". If this link does not appear, there are no attachment files.]

To ensure your figures meet our technical requirements, please review our figure guidelines: https://journals.plos.org/plosone/s/figures

You may also use PLOS’s free figure tool, NAAS, to help you prepare publication quality figures: https://journals.plos.org/plosone/s/figures#loc-tools-for-figure-preparation.

NAAS will assess whether your figures meet our technical requirements by comparing each figure against our figure specifications.

# Author comment: Author response to Decision Letter 1

**DOI:** 10.1371/journal.pone.0357478.r002

7 Aug 2026

August 3, 2026

Dr. Vasileios Kallinterakis

Academic Editor - PLOS One

Dear Dr. Kallinterakis -

Thanks for the prompt review of our manuscript “The Well-Being of Jewish College Students in the United States (PONE-D-26-20428).” Drawing on the helpful recommendations from reviewers, we revised the paper extensively, and believe it has improved considerably as a result. In the letter below, we respond point-by-point to your comments and those of the reviewers.

Editor’s Comments

Comment 1 - Style

Please ensure that your manuscript meets PLOS ONE's style requirements.

Response 1 - Style

We reviewed the style templates and revised the submission accordingly.

Comment 2 - Code

Please review our guidelines ... and ensure that your code is shared in a way that follows best practice and facilitates reproducibility and reuse.

Response 2 - Code

We read the guidelines carefully, and don’t believe that in our paper “author-generated code underpins the findings.” Of course, we could be mis-interpreting the guidelines. If there’s specific code you have in mind - e.g., syntax used for statistical analysis - we’re happy to post it on OSF along with the pre-registration and survey materials.

Comment 3 - Acknowledgments

Please remove any funding-related text from the manuscript and let us know how you would like to update your Funding Statement.

Response 3 - Acknowledgments

Sorry for the mistakes. We removed the Acknowledgement section from the manuscript, since there’s no relevant material to place there. And here’s an updated funding statement, which contains all the information you requested.

“This research was supported by grants from an Anonymous foundation (to GEM, #2024-5981)

and Northwestern University (to SIL, #10029690). The funders had no role in the design, analysis, or writing of study.”

Comment 4 - Funding Statement

We note that the grant information you provided in the ‘Funding Information’ and ‘Financial Disclosure’ sections do not match.

Response 4 - Funding Statement

Again, sorry about the mistake - see response above for an updated funding statement. We have added this material to the Funding Disclosure section in the manuscript portal.

Comment 5 - Supporting Info

Please include captions for your Supporting Information files at the end of your manuscript, and update any in-text citations to match accordingly.

Response 5 - Supporting Info

We updated the manuscript as directed, see p. 33.

Comment 6 - Citations

If the reviewer comments include a recommendation to cite specific previously published works, please review and evaluate these publications to determine whether they are relevant and should be cited. There is no requirement to cite these works unless the editor has indicated otherwise.

Response 6 - Citations

Thanks for the clarification on this issue.

Reviewer #1

Comment 1 - Abstract

-Line 31 “To fill these gaps in knowledge.” remove period which appears mid sentence.

-Lines 31-32 Unclear how student well-being one year later (January-June 2025) exactly relates to student well-being a year earlier when Israel-Hamas protests/conflict-related incidents were at their peak (Spring 2024). Geopolitical events and climate shift rapidly- a year later, the climate may not be ideal for measuring how students experienced the aforementioned events. There needs to be a bit more rationale here as to the time lag. Anti-semitism is ongoing- but the authors mention the events of October 7 and clearly highlight spring 2024. Yet the data timeline does not align with that.

-Line 35 Second aim is lengthy/reads unwieldy. Perhaps streamline to support the readability.

-Specific survey content including tools used not specified in Abstract, limiting the reader’s understanding of the scope of data collection. This can be added in succinctly/briefly for clarification.

Response 1 - Abstract

Thanks. We’ve incorporated these helpful suggestions into the revised abstract.

Comment 2 - Introduction

-Lines 64-65 “ Nearly one-third” - report or reported? The rest of this paragraph is past tense.

-Lines 67-70 Sentence structure does not make sense. Please check.

-Lines 73-74 Needs its’ own citations - UCLA task force is not (alone) ‘policymakers and politicians’ as stated.

-Lines 79-83 It would be helpful to foreshadow some of the mentioned ‘range of protective factors’ as these are relevant later on in the paper.

-Lines 84-94 This paragraph is clear and timely- however racially minoritized youth is not mentioned previously- and this bears mention since Jewish students are a sub-group of this- and previous research does exist within other racially minoritized student groups- relevant to this study.

Response 2 - Introduction

Again, thanks for the helpful feedback. We have extensively revised the Introduction, incorporating these suggestions along with recommendations from the other reviewer.

Comment 3 - Methods and Materials

- Lines 135-149 Were these questions asked only of Jewish students?

- Line 151-159 Health Minds Study requires citation

- Line 175 (179) How was neuroticism assessed? You mention a 3 item scale, but the flow of this section is disjointed. I suggest a clear flow where the entire survey content, who receive which questions etc. is included, rather than a narrative which seems to switch back and forth between survey content and data analysis. (You may include this in a chart/table for clarity.)

Response 3 - Methods and Materials

Making these changes has improved the clarity of the section. At the outset of the measures section, we now provide a hyperlink to the full text of the survey (line 148) and state that unless otherwise specified, all instruments were administered to all participants (lines 148-150). We also provide citations for papers and manuals relevant to the Healthy Minds Study (line 192) and clarify how neuroticism was measured (lines 219-220).

Comment 4 - Positionality Statement

No positionality statements are included. Given the subject matter of this manuscript, strongly suggest to include. Topics such as Jewish identity, belonging, campus climate and Israel-Hamas conflict are politically and emotionally charged. Why are the authors uniquely qualified to study this, not only from a professional perspective but also from a personal one? If not uniquely qualified, please provide some context as to background/potential bias. This information provides context.

Response 4 - Positionality Statement

Although all of the authors of the study identify as Jewish and are concerned about rising antisemitism in the US, we have chosen not to include a positionality statement, as we believe it is not warranted for the current study. In qualitative data collection, positionality statements can be helpful, because (for instance), in conducting interviews and field observations, the researcher is the instrument of data collection. Data are produced through interactions involving the researcher herself. Moreover, the interpretive and iterative nature of qualitative data analysis means that a researcher’s personal biography and concerns can shape which findings she chooses to make central and which are more peripheral. The current study, by contrast, uses standardized survey instruments. Moreover, the surveys were not administered by any particular individual; participants responded to survey questions on an online platform. Finally, we preregistered our hypotheses and analytic procedures to guard against potential biases and dispositions that might shape our interpretation of the results.

Comment 5 - Results

Line 270 “Those who are Jewish” rather state “Those who identified as Jewish”

Response 5 - Results

We have updated this language. Also, given the other reviewer’s comments regarding this issue, we have explained in multiple places that our categorization is based on students’ religious identity.

Comment 6 - Discussion

Discussion needs to be situated in more literature (and add this to introduction as well). Also, can split up with/without subheadings into Discussion, Limitations, Implications, Conclusion. Also, please organize the content to flow with longer, more coherent paragraphs.

Response 6 - Discussion

Thanks for these helpful suggestions. We substantially revised the content and structure of the Discussion, guided by these suggestions and those from the other reviewer.

Reviewer #2

Comment 1 - General

Thank you for this interesting and timely study examining Jewish student well-being in the post-October 7th campus climate. The topic is important and the data have the potential to make a meaningful contribution to the literature. However, I have identified several methodological and framing concerns that I believe must be addressed to strengthen the paper for publication.

Response 1 - General

Thanks for the kind words about the paper.

Comment 2 - Preregistration

The preregistration was not available on OSF—only the survey was uploaded. Please make the preregistration publicly available so that adherence to the preregistered analytic plan can be evaluated.

Response 2 - Preregistration

We apologize for this mistake. The revised manuscript provides the correct hyperlink to the preregistration materials (line 129).

Comment 3 - Introduction

The introduction would benefit from a brief description of the events of October 7th (i.e., the terrorist attack and ensuing war) to contextualize the study for readers unfamiliar with the timeline. Consider noting that upticks in antisemitism are often reported globally following Israeli military action, as this provides important historical context. Relatedly, the statement "encampments that centered Zionism and the Israeli government" is vague and could be elaborated to more fully capture the nuance of the encampment formation.

The introduction should also discuss how the US government response differed from that of other countries—where protests and encampments were largely permitted to continue—making the US a unique case study.

The study's framing would benefit from significant revision to reflect the timeline of data collection with reference to sociopolitical events. Your data capture Jewish student well-being after substantial governmental and institutional attention had been given to the upsurge in antisemitism on college campuses. As such, the paper cannot be characterized as an examination of heightened antisemitism on college campuses without the important contextual detail that institutions had, often as a result of direct or indirect action from the government, implemented policies or procedures in an attempt to address Jewish students' civil rights. This temporal and policy context may explain why findings diverge from other studies conducted during this period and should be foregrounded throughout.

Please include specific hypotheses in the introduction, including hypotheses for the HMS data, whose inclusion is otherwise unclear.

Response 3 - Introduction

Thanks for the helpful suggestions. We’ve incorporated them - along with suggestions from Reviewer 1 - into the revision. As a result of these changes, the Introduction has improved considerably. The only recommendation we’ve passed on is the one to include specific hypotheses. As the pre-registration materials indicate, we did not begin the study with “strong a priori hypothesis about the well-being of Jewish students relative to their peers of other religious identities. ... our goal (was) not to test a specific hypothesis, but instead to accurately describe the well-being of Jewish students, by accruing a large, diverse sample...” With that said, we have added material to the Introduction clarifying the rationale for HMS analyses (lines 114-118). Finally, the point about the US as a “unique case study” is an important one. We now raise it in the section of the Discussion about generalizability, where it ties in with other concerns about what our sample can - and can’t - tell readers about the well-being of Jewish students (lines 455-458).

Comment 4: Measures & Survey Design

Judaism is framed as a religious identity, which is a notable limitation. Most recent studies (e.g., your reference #26) avoid this framing. Many Jews identify as atheist or agnostic, and this group may be particularly affected by campus climate through lower levels of connection to the Jewish community via institutionalized religious practice. Please make explicit throughout the paper that you are assessing Judaism in the religious sense and not as an ethnicity, and discuss how this framing may have impacted your data and results.

Response 4: Measures & Survey Design

This is an important point. We highlight it in the Methods (lines 156-159), where the approach to measuring religion is introduced, and again In the Discussion, where we consider how it may have contributed to the results (lines 451-455).

Comment 5 - Other Responses

Most demographic items appear to have included an open-ended response option in the survey uploaded to OSF. Was this also the case for religion? If not, please explain the rationale. If so, elaborate on how "other" responses were coded.

Response 5 - Other Responses

Unlike the other demographic items, the question about religion item did not provide an option for an open-ended response. This was a clerical error on our part. However, this item did have the response option “Other religion not specified.” As we now state on line 162, students who selected this option were placed in the “Other Religion” category.

Comment 6 - Jewish ethnicity

For the race/ethnicity item, did any respondents identify as Jewish (or Ashkenazi, Sephardic, Mizrahi)? If so, how were these responses handled? This relates directly to the concern about respondents who identify as ethnically but not religiously Jewish.

Response 6 - Jewish ethnicity

No students responded in this way.

Comment 7 - Gender

Please clarify how gender was coded. The paper states that "female" was used as the reference category, but the survey uses the term "woman" and includes six other gender categories. Collapsing men with all other categories makes little theoretical sense. Please explain and justify the coding scheme.

Response 7 - Gender

These are good questions and we apologize for the confusion - our bad. We’ve updated the paper to clarify our rationale and approach to modeling gender (see lines 213-217). We opted to code gender into two categories (female vs. other) to harmonize our survey with results from HMS, which until the later years did not have more granular response options. To make sure this approach didn’t bias our results, we reconducted the primary analyses after excluding 84 students in our survey (2.4%) whose responses were not either “man” or “woman.” The findings were identical. Throughout the paper and tables, we have replaced “female” with “woman.” Thanks for catching this.

Comment 8 - HMS

Please describe how religious identity is assessed in the HMS study, and how HMS demographic covariates were assessed and may differ from those in the present study.

Response 8 - HMS

We now provide more detail about these items on lines 192-199. We also note in the Discussion that variations in the wording/format of these items may have contributed to the slightly different patterns of results observed in the two studies (lines 471-473).

Comment 9 - Campus atmosphere

Please elaborate on how the scale to capture campus atmosphere was created and how items were selected.

Response 9 - Campus atmosphere

We added material on these items to the Methods, see lines 177-187.

Comment 10 - Error describing item

There appears to be an error in the scale items listed at lines 144–147. I believe the first item should be "In order to fit in on my campus, I feel the need to hide that I am Jewish," with the remaining items following accordingly. Please verify and correct.

Response 10 - Error describing item

Thanks for catching this mistake. The section now reads as it should (lines 182-183).

Comment 11 - Misc suggestions

- Please include how neuroticism was assessed in the methods section.

- Cronbach's "a" should be "α" throughout.

- Line 172: "ethnic identify" should be "ethnic identity."

Response 12 - Misc sugg

Attachment Submitted filename: PLOS One response letter.docx

# Peer review: Decision Letter 1

**DOI:** 10.1371/journal.pone.0357478.r003

**Authors:** Vasileios Kallinterakis

18 Aug 2026

The Well-Being of Jewish College Students in the United States

PONE-D-26-20428R1

Dear Dr. Miller,

We’re pleased to inform you that your manuscript has been judged scientifically suitable for publication and will be formally accepted for publication once it meets all outstanding technical requirements.

Within one week, you’ll receive an e-mail detailing the required amendments. When these have been addressed, you’ll receive a formal acceptance letter and your manuscript will be scheduled for publication.

An invoice will be generated when your article is formally accepted. Please note, if your institution has a publishing partnership with PLOS and your article meets the relevant criteria, all or part of your publication costs will be covered. Please make sure your user information is up-to-date by logging into Editorial Manager at Editorial Manager® and clicking the ‘Update My Information' link at the top of the page. For questions related to billing, please contact billing support.

If your institution or institutions have a press office, please notify them about your upcoming paper to help maximize its impact. If they’ll be preparing press materials, please inform our press team as soon as possible -- no later than 48 hours after receiving the formal acceptance. Your manuscript will remain under strict press embargo until 2 pm Eastern Time on the date of publication. For more information, please contact onepress@plos.org.

Kind regards,

Vasileios Kallinterakis

Academic Editor

PLOS One

Additional Editor Comments (optional):

Reviewers' comments:

Reviewer's Responses to Questions

**Comments to the Author**

Reviewer #1: All comments have been addressed

Reviewer #2: All comments have been addressed

**********

2. Is the manuscript technically sound, and do the data support the conclusions?

Reviewer #1: Yes

Reviewer #2: Yes

**********

3. Has the statistical analysis been performed appropriately and rigorously?

Reviewer #1: Yes

Reviewer #2: Yes

**********

4. Have the authors made all data underlying the findings in their manuscript fully available?

The PLOS Data policy

Reviewer #1: Yes

Reviewer #2: No

**********

5. Is the manuscript presented in an intelligible fashion and written in standard English?

Reviewer #1: Yes

Reviewer #2: Yes

**********

Reviewer #1: The authors are commended for a much improved and clear manuscript. My only comment would be that my preference is a Discussion section that includes subheadings (this one was continuous, without subheadings). I anticipate that this study will make a significant contribution to the literature, and have an impact on how university-student mental health/wellbeing is understood.

Reviewer #2: The authors have adequately addressed my comments on their prior draft. I look forward to seeing the manuscript published.

**********

what does this mean?). If published, this will include your full peer review and any attached files.

If you choose “no”, your identity will remain anonymous but your review may still be made public.

**Do you want your identity to be public for this peer review?**  For information about this choice, including consent withdrawal, please see our Privacy Policy

Reviewer #1: **Yes:** Zahava L. Friedman

Reviewer #2: No

**********

# Editor report: Acceptance letter

**DOI:** 10.1371/journal.pone.0357478.r004

**Authors:** Vasileios Kallinterakis

PONE-D-26-20428R1

PLOS One

Dear Dr. Miller,

I'm pleased to inform you that your manuscript has been deemed suitable for publication in PLOS One. Congratulations! Your manuscript is now being handed over to our production team.

At this stage, our production department will prepare your paper for publication. This includes ensuring the following:

* All references, tables, and figures are properly cited

* All relevant supporting information is included in the manuscript submission,

* There are no issues that prevent the paper from being properly typeset

You will receive further instructions from the production team, including instructions on how to review your proof when it is ready. Please keep in mind that we are working through a large volume of accepted articles, so please give us a few days to review your paper and let you know the next and final steps.

Lastly, if your institution or institutions have a press office, please let them know about your upcoming paper now to help maximize its impact. If they'll be preparing press materials, please inform our press team within the next 48 hours. Your manuscript will remain under strict press embargo until 2 pm Eastern Time on the date of publication. For more information, please contact onepress@plos.org.

You will receive an invoice from PLOS for your publication fee after your manuscript has reached the completed accept phase. If you receive an email requesting payment before acceptance or for any other service, this may be a phishing scheme. Learn how to identify phishing emails and protect your accounts at https://explore.plos.org/phishing.

If we can help with anything else, please email us at customercare@plos.org.

Thank you for submitting your work to PLOS One and supporting open access.

Kind regards,

PLOS One Editorial Office Staff

on behalf of

Dr. Vasileios Kallinterakis

Academic Editor

PLOS One
